# *Mycobacterium tuberculosis* produces d-serine under hypoxia to limit CD8^+^ T cell-dependent immunity in mice

**DOI:** 10.1038/s41564-024-01701-1

**Published:** 2024-05-28

**Authors:** Hongyu Cheng, Zhe Ji, Yang Wang, Shenzhi Li, Tianqi Tang, Fei Wang, Cheng Peng, Xiangyang Wu, Yuanna Cheng, Zhonghua Liu, Mingtong Ma, Jie Wang, Xiaochen Huang, Lin Wang, Lianhua Qin, Haipeng Liu, Jianxia Chen, Ruijuan Zheng, Carl G. Feng, Xia Cai, Di Qu, Lilin Ye, Hua Yang, Baoxue Ge

**Affiliations:** 1 Shanghai Key Laboratory of Tuberculosis, Shanghai Pulmonary Hospital, Tongji University School of Medicine, Shanghai, P. R. China; 2 Department of Microbiology and Immunology, Tongji University School of Medicine, Shanghai, PR China; 3 Clinical and Translational Research Center, Shanghai Pulmonary Hospital, Tongji University School of Medicine, Shanghai, P. R. China; 4 Immunology and Host Defense Group, Faculty of Medicine and Health, The University of Sydney, Sydney, New South Wales Australia; 5 Tuberculosis Research Program, Centenary Institute, The University of Sydney, Sydney, New South Wales Australia; 6 Biosafety Level 3 Laboratory, Shanghai Medical College, Fudan University, Shanghai, P. R. China; 7 Institute of Immunology, Third Military Medical University, Chongqing, P. R. China

**Keywords:** Cellular microbiology, Lymphocyte activation

## Abstract

Adaptation to hypoxia is a major challenge for the survival of *Mycobacterium tuberculosis* (*Mtb*) in vivo. Interferon (IFN)-γ-producing CD8^+^ T cells contribute to control of *Mtb* infection, in part by promoting antimicrobial activities of macrophages. Whether *Mtb* counters these responses, particularly during hypoxic conditions, remains unknown. Using metabolomic, proteomic and genetic approaches, here we show that *Mtb* induced Rv0884c (SerC), an *Mtb* phosphoserine aminotransferase, to produce d-serine. This activity increased *Mtb* pathogenesis in mice but did not directly affect intramacrophage *Mtb* survival. Instead, d-serine inhibited IFN-γ production by CD8^+^ T cells, which indirectly reduced the ability of macrophages to restrict *Mtb* upon co-culture. Mechanistically, d-serine interacted with WDR24 and inhibited mTORC1 activation in CD8^+^ T cells. This decreased T-bet expression and reduced IFN-γ production by CD8^+^ T cells. Our findings suggest an *Mtb* evasion mechanism where pathogen metabolic adaptation to hypoxia leads to amino acid-dependent suppression of adaptive anti-TB immunity.

## Abstract (web summary)

Hypoxia induces d-serine production by *Mycobacterium tuberculosis* which inhibits mTORC1 and T-bet expression to restrict downstream CD8^+^ T cell immune responses and macrophage activation, and inhibit control of tuberculosis in mice.

## Main

*Mycobacterium tuberculosis* (*Mtb*) is an extremely successful intracellular pathogen that resides in host macrophages. Macrophages, lymphocytes and other leucocytes coalesce to form the classical tuberculous granuloma, a cellular environment that is believed to be hypoxic^1–3^. Cellular metabolism plays an important role in immune responses to *Mtb* infection. Macrophages respond to infection by profoundly changing their metabolism. *Mtb* appears to take advantage of the changes to its benefit^4^. Studies have shown that *Mtb* depends on the asparagine transporter AnsP2 to capture asparagine from macrophages, and asparagine reacts with protons in the phagosome to resist acid stress^5^. l-arginine is essential for macrophages to generate nitrogen monoxide (NO) through inducible Nitric Oxide Synthase (iNOS)^6^. This process can be interrupted by type 1 arginase (Arg1), which is induced in human macrophages within tuberculous granulomas and in lung tissues of mice infected with Bacille Calmette–Guérin (BCG)^6^.

Adaptation to hypoxia is one of the major challenges for *Mycobacteria* to maintain successful persistent infection in the granulomas^1–3^ or inside macrophages^7^. Our previous work has shown that *Mycobacteria* under hypoxia secrete the protein Rv0859/MMAR_4677 (Fatty-acid degradation A, FadA) that modulates the host fatty acid metabolism to suppress H3K9Ac-mediated expression of the host proinflammatory cytokine *Il6*, thus promoting granuloma progression^8^. Hypoxia induces widespread transcriptional changes of mycobacterial genes associated with a metabolically altered state^9^. However, whether and how hypoxia induces these metabolic changes in *Mtb* to integrate hypoxia adaptation with inhibition of host anti-tuberculosis (TB) immunity remain unclear.

An effective adaptive immune response is required to prevent progressive, disseminated TB. A major function of T cells in *Mtb* control is cytokine production. CD8^+^ or CD4^+^ T cells respond to *Mtb* by producing IFN-γ that is critical for the control of *Mtb* infection by stimulating macrophages^10–14^. Although CD4^+^ T cells are thought to be more important, there is increasing evidence for a role of CD8^+^ T cells in the control of *Mtb* infection. In a non-human primate (NHP) infection model, CD8^+^ T cell responses were proven essential against acute and chronic *Mtb* infection, and correlated with protection elicited by BCG vaccination^13,15–17^. In another mouse model study, control of latent but not active *Mtb* infection was reportedly dependent on CD8^+^ T cells^18^. Notably, antigen-specific CD8^+^ T cells poorly recognize *Mtb-*infected macrophages^19,20^, indicating an unexplored potential for impairment of CD8^+^ T cells by *Mtb*.

Here we show that *Mtb* induces the expression of a phosphoserine aminotransferase, Rv0884c (SerC), to increase d-serine production under hypoxic conditions. d-serine suppresses CD8^+^ T cell-dependent interactions with macrophages and macrophage control of *Mtb* by inhibiting IFN-γ production. We provide molecular and genetic evidence demonstrating that d-serine, which directly interacts with WD repeat-containing protein 24 (WDR24), inactivates the mechanistic target of rapamycin complex 1 (mTORC1) pathway to reduce the generation of IFN-γ-producing CD8^+^ T cells.

## Results

### Hypoxia induces d-serine production by *Mtb*

**Fig. 1:**
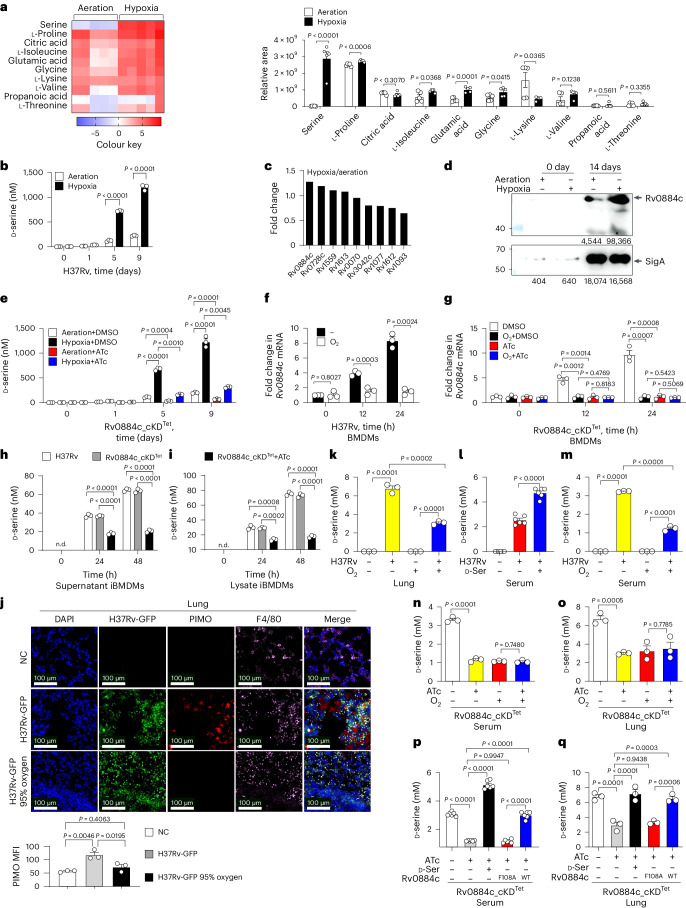
Mycobacterial d-serine is induced by hypoxia through Rv0884c. **a**, Heat map (left) and relative area (right) of part of the metabolite differentially secreted from H37Rv incubated under aeration or hypoxia for 14 days. **b**, Assay of d-serine concentration from culture supernatants of H37Rv incubated under aeration and hypoxia for indicated days. **c**, Fold change of serine metabolism-related proteins from proteomic profiling of cell lysates from H37Rv under aeration or hypoxia for 14 days. **d**, Immunoblot (IB) of cell lysate from H37Rv incubated under aeration and hypoxia. Levels of Rv0884c and SigA proteins were quantified using ImageJ and are indicated below blots. **e**, d-serine concentration in the culture supernatants from Rv0884c_cKD^Tet^ strain incubated with or without ATc under aeration and hypoxia for indicated days. **f**,**g**, qPCR analysis of *Rv0884c* mRNA from BMDMs infected with H37Rv (**f**) or Rv0884c_cKD^Tet^ strain treated with DMSO or ATc (**g**) and incubated in the condition of normal air (−) or 95% O_2_ (O_2_) for indicated times. **h**,**i**, d-serine concentration in the culture supernatants (**h**) or cell lysates (**i**) from iBMDMs infected with H37Rv or Rv0884c_cKD^Tet^ strain incubated with or without ATc for indicated times. n.d., not detected. **j**–**o**, C57BL/6 mice were aerosol-infected with GFP-H37Rv (**j**,**k**,**m**), H37Rv (**l**) or Rv0884c_cKD^Tet^ and then fed with or without doxycycline (**n**,**o**) for 4 weeks. Infected mice were exposed to either 95% O_2_ or normal air for 20 h before being killed. NC, uninfected mice. Lung immunofluorescence staining of PIMO (red), H37Rv (green), F4/80 (pink) and DAPI (blue) was performed. Mean fluorescence intensity (MFI) of PIMO was quantified using ImageJ (**j**). d-serine concentration in the supernatant of lung homogenates (**k**,**o**) and serum (**l**–**n**) was analysed. **p**,**q**, C57BL/6 mice were aerosol-infected with indicated *Mtb* strains for 4 weeks. d-serine concentration in serum (**p**) and lung homogenate supernatant (**q**) was analysed. Data in **a**, **b** and **d**–**q** represent one experiment with at least three independent biological replicates. Results are shown as mean ± s.e.m. Two-tailed unpaired Student’s *t*-test (**a**,**b**,**e**–**i**,**k**–**o**) and one-way ANOVA with Tukey’s multiple comparisons test (**j**,**p**,**q**) were used for statistical analysis. Source data

**Extended Data Fig. 1:**
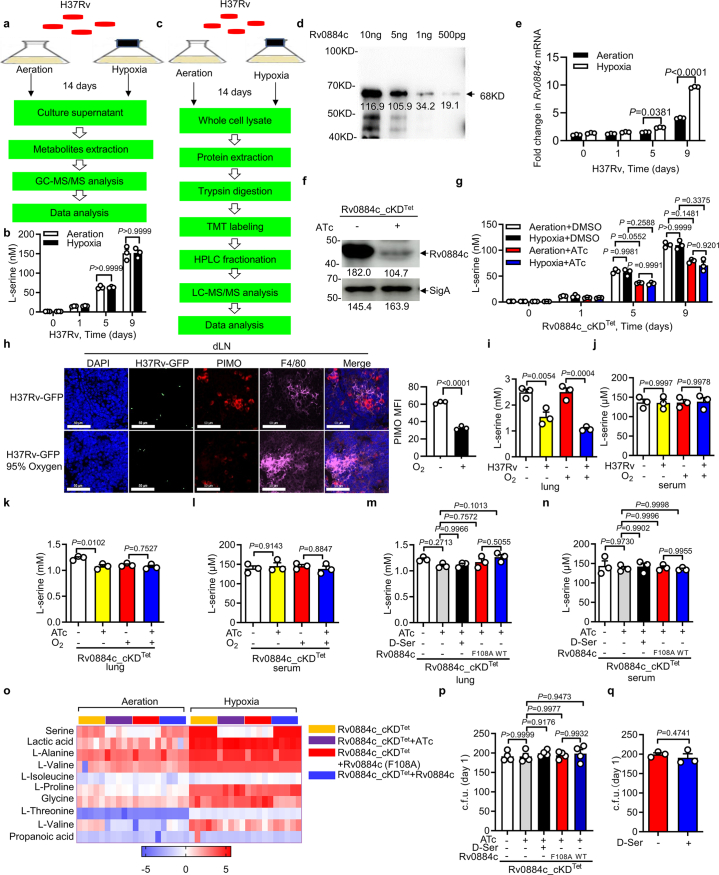
Mycobacterial D-serine, but not L-serine is induced by hypoxia through Rv0884c. (**a**) Quantitative metabolomics approach. (**b**) Assay of L-serine concentration from culture supernatants of H37Rv incubated under aeration or hypoxia. (**c**) Quantitative proteomics approach. (**d**) IB of Rv0884c_107-376aa_. (**e**) qPCR analysis of *Rv0884c* mRNA from H37Rv incubated under aeration or hypoxia. (**f**) IB of cell lysates derived from Rv0884c_cKD^Tet^ strains treated with or without ATc. (**g**) L-serine concentration in the culture supernatants from Rv0884c_cKD^Tet^ strain incubated with or without ATc under aeration or hypoxia. (**h-j**) Mice infected with GFP-H37Rv for 4 weeks were exposed to either 95% O_2_ or normal air for 20 hours before sacrifice. Immunofluorescence staining of dLN was performed. MFI of PIMO were quantified with ImageJ (**h**). Scale bar, 100 μm; L-serine concentration of lung homogenate supernatants (**i**) and sera (**j**) were analyzed. (**k, l**) Mice infected with Rv0884c_cKD^Tet^ treated with or without ATc for 4 weeks were exposed to either 95% O_2_ or normal air for 20 hours before sacrifice. L-serine concentration of lung homogenate supernatant (**k**) and serum (**l**) were analyzed. (**m-n, p**) L-serine concentration of lung homogenate supernatant (**m**) and serum (**n**) from indicated *Mtb* strains infected-mice were analyzed at 4 weeks post-infection; The c.f.u. of lungs were assayed 1 day post-infection (**p**). (**o**) Heatmap of metabolites secreted from indicated *Mtb* strains incubated under aeration or hypoxia. (**q**) H37Rv infected-mice were administered with or without D-serine. Lung c.f.u. were assayed 1 day post-infection. All data represent one experiment with at least three independent biological replicates. Results are shown as mean ± s.e.m. Two-way ANOVA with Tukey’s multiple comparisons test (**b, e, g**), one-way ANOVA with Tukey’s multiple comparisons test (**i-n, p**) and Two-tailed unpaired Student’s t-test (**h**, **q**) were used for statistical analysis. Source data

To determine the metabolic responses of *Mycobacteria* to hypoxia, virulent *Mtb* H37Rv was cultured under hypoxic conditions using Wayne’s in vitro hypoxia model^21,22^, in which *Mtb* was grown in sealed tubes and the gradual depletion of oxygen resulted in a hypoxic non-replicating persistence of *Mtb*^8^; comparative metabolomics analysis of culture supernatants (Extended Data Fig. 1a) revealed that hypoxia significantly increased the levels of serine (Fig. 1a and Supplementary Table 1). By using the d-serine or l-serine assay kit^23^, we confirmed that d-serine levels (Fig. 1b), but not l-serine levels (Extended Data Fig. 1b), were significantly increased in the culture filtrates from *Mtb* H37Rv under hypoxia. In addition, proteomics analysis of cell lysates from *Mtb* H37Rv (Extended Data Fig. 1c and Supplementary Table 2) showed that the protein level of an *Mtb* phosphoserine aminotransferase, Rv0884c (PSAT or SerC; EC 2.6.1.52), was increased under hypoxia (Fig. 1c), which was further confirmed by western blot analysis using an anti-Rv0884c polyclonal antibody (Fig. 1d and Extended Data Fig. 1d). Similarly, the messenger RNA level of *Rv0884c* was significantly increased under hypoxia (Extended Data Fig. 1e).

As Rv0884c is an essential gene for the viability of the cell^24,25^, we used a well-established CRISPR interference method^26^ to generate an anhydrotetracycline (ATc)-inducible, Rv0884c-knockdown (Rv0884c_cKD^Tet^) *Mtb* H37Rv strain (Extended Data Fig. 1d,f). ATc-induced knockdown of Rv0884c in *Mtb* H37Rv reduced the basal secretion of d-serine and l-serine under both aeration and hypoxia (Fig. 1e and Extended Data Fig. 1g), but only significantly downregulated the hypoxia-induced increase in secretion of d-serine, not l-serine (Fig. 1e and Extended Data Fig. 1g).

The intracellular environment of *Mtb*-infected macrophages is hypoxic even when macrophages are incubated under standard tissue culture conditions^7^. Accordingly, bone marrow-derived macrophages (BMDMs) infected with H37Rv showed significantly increased level of *Rv0884c* mRNA (Fig. 1f). However, this increase was not observed when H37Rv-infected BMDMs were exposed to 95% O_2_ through the addition of carbogen, which can reverse the hypoxic condition^3^ (Fig. 1f). In addition, BMDMs infected with ATc-treated Rv0884c_cKD^Tet^ strain displayed significantly decreased level of *Rv0884c* mRNA compared with its non-treated counterpart; however, there was no difference in induction when ATc-treated and non-treated Rv0884c_cKD^Tet^-infected BMDMs were exposed to 95% O_2_ (Fig. 1g). Levels of d-serine were significantly increased in immortalized BMDMs (iBMDMs) infected with H37Rv or non-treated Rv0884c_cKD^Tet^ strain, but impaired in cells infected with ATc-induced knockdown mutant (Fig. 1h,i).

*Mtb* infection disseminates from the distal airways to the lung tissues^2^; then *Mtb*-infected macrophages traffic to the lung-draining lymph nodes (dLNs) to prime T cells^1–3^. To confirm whether hypoxia induces Rv0884c and serine production during in vivo infection, the oxygen tension in the lung tissues and dLNs of C57BL/6 mice was assessed using pimonidazole (PIMO) staining. Both the lung tissues and dLNs of GFP-H37Rv-infected C57BL/6 mice were hypoxic according to positive PIMO staining signals^7,27^, which significantly decreased after exposure to 95% O_2_ (Fig. 1j and Extended Data Fig. 1h). Consistently, the level of d-serine, but not l-serine, was significantly increased in the lung tissues and serum from C57BL/6 mice infected with H37Rv (Fig. 1k–m and Extended Data Fig. 1i,j). However, exposure of the GFP-H37Rv-infected mice to 95% O_2_ eliminated the increase in d-serine levels in lung tissues and serum (Fig. 1k,m). Furthermore, knockdown of Rv0884c significantly reduced the level of d-serine, but not l-serine, in serum and lung tissues of H37Rv-infected mice (Fig. 1n–q and Extended Data Fig. 1k–n). Increased levels of d-serine were not observed in the non-ATc-treated Rv0884c_cKD^Tet^-infected mice exposed to 95% O_2_ (Fig. 1n,o).

Previous structural analysis of *Mtb* Rv0884c has identified a key aminotransferase enzymatic site, F108 (refs. ^28,29^). The F108A mutants of the aminotransferase enzyme site in Rv0884c exhibited a decrease in enzymatic activity compared with wild-type (WT) Rv0884c^28,29^. We constructed the WT-complemented H37Rv (Rv0884c_cKD^Tet^+Rv0884c) strain and a mutant-complemented H37Rv (Rv0884c_cKD^Tet^+Rv0884c (F108A)) strain, in which the catalytic activity is abrogated. Metabolomic analysis of culture supernatants revealed that complementation with WT Rv0884c, but not with (F108A)-mutant Rv0884c could restore serine production by the Rv0884c-knockdown strain under hypoxia (Extended Data Fig. 1o and Supplementary Table 3). Similarly, increased levels of d-serine but not l-serine were detected in the serum and lung tissues of mice infected with the Rv0884c-knockdown strain complemented with WT Rv0884c, but not with Rv0884c (F108A) (Fig. 1p,q and Extended Data Fig. 1m,n).

### d-serine impairs CD8^+^ T cell-mediated anti-TB immunity

**Fig. 2:**
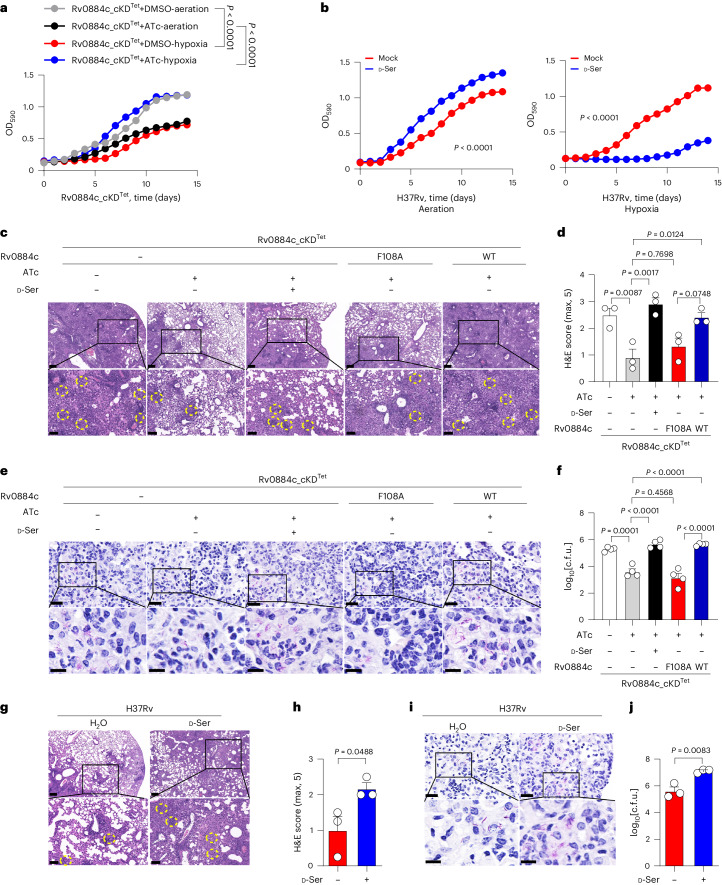
Rv0884c/d-serine impairs anti-TB immunity. **a**, In vitro growth curve of Rv0884c_cKD^Tet^ incubated with or without ATc under aeration or hypoxia at 37 °C for 14 days. **b**, In vitro growth curve of H37Rv with addition of 10 mM d-serine incubated under aeration (left) or hypoxia (right) at 37 °C for 14 days. **c**–**f**, C57BL/6 mice were aerosol-infected with ~200 c.f.u. per mouse of Rv0884c_cKD^Tet^ with or without ATc, Rv0884c_cKD^Tet^ with ATc and d-serine, Rv0884c_cKD^Tet^+Rv0884c (F108A) with ATc, and Rv0884c_cKD^Tet^+Rv0884c with ATc. After 4 weeks of infection, histopathology of lung sections from infected mice was assessed via H&E staining (**c**; scale bar, 200 μm (top) and 100 μm (bottom)), histology score (**d**), acid-fast staining (**e**; scale bar, 20 μm (top) and 10 μm (bottom)) and bacterial load (**f**). The areas of leucocyte infiltration between conditions are labelled using yellow dotted circles. **g**–**j**, C57BL/6 mice were aerosol-infected with ~200 c.f.u. per mouse of H37Rv, and then 30 g l^−1^ d-serine was administered via drinking water to half of the infected mice. After 4 weeks of infection, histopathology of lung sections from infected mice was assessed via H&E staining (**g**; scale bar, 200 μm (top) and 100 μm (bottom)), histology score (**h**), acid-fast staining (**i**; scale bar, 20 μm (top) and 10 μm (bottom)) and bacterial load (**j**). The areas of leucocyte infiltration between conditions are labelled using yellow dashed circles. Data in **a**–**j** represent one experiment with at least three independent biological replicates. Results are shown as mean ± s.e.m. Two-way ANOVA with Tukey’s multiple comparisons test (**a**,**b**), one-way ANOVA with Tukey’s multiple comparisons test (**d**,**f**) and two-tailed unpaired Student’s *t*-test (**h**,**j**) were used for statistical analysis. Source data

Conditional knockdown of Rv0884c reduced the in vitro growth of *Mtb* H37Rv under aeration (Fig. 2a). However, under hypoxic conditions, knockdown of Rv0884c substantially increased in vitro growth of H37Rv, while supplementation with d-serine reduced growth (Fig. 2a,b). We next tested the contribution of Rv0884c to *Mtb* infection in vivo. Mice infected with Rv0884c_cKD^Tet^ complemented with Rv0884c had more severe pathology and significantly higher bacterial burdens in their lung tissues than mice infected with Rv0884c-knockdown strain, or those infected with the Rv0884c (F108A) complemented strain (Extended Data Fig. 1p and Fig. 2c–f). Similarly, treatment of *Mtb*-infected mice with d-serine led to more severe pathology and significantly higher bacterial burden in lung tissues (Fig. 2g–j and Extended Data Fig. 1q).

**Fig. 3:**
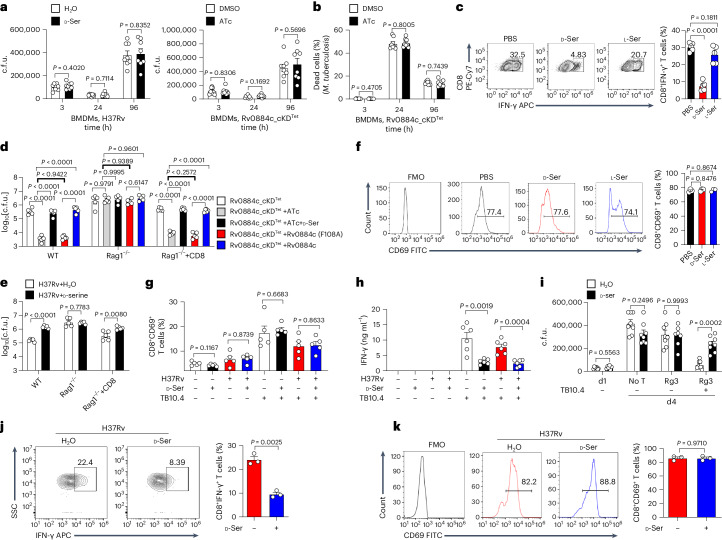
d-serine inhibits IFN-γ production by CD8^+^ T cells. **a**, Intracellular survival of H37Rv in BMDMs treated with or without d-serine (10 mM) (left) or Rv0884c_cKD^Tet^ treated with or without ATc in BMDMs (right) was assessed using c.f.u. assay. **b**, Bacterial viability of Rv0884c_cKD^Tet^ treated with or without ATc in BMDMs was assayed using the LIVE/DEAD BacLight bacterial viability kit. **c**,**f**, Naïve CD8^+^ T cells were activated and differentiated with anti-CD3 and anti-CD28 antibodies in the presence of IL-2 and IL-12 p70 for 5 days and treated with PBS, d-serine or l-serine. Percentage of IFN-γ^+^ (**c**) and CD69^+^ (**f**) CD8^+^ T cells was assayed. APC, allophycocyanin. **d**,**e**, WT mice, *Rag1*^−/−^ mice and *Rag1*^−/−^ mice adoptively transferred with CD8^+^ T cells (*Rag1*^−/−^+CD8) were aerosol-infected with ~200 c.f.u. of indicated *Mtb* strains (**d**, Rv0884c_cKDTet with or without ATc, Rv0884c_cKDTet+Rv0884c (F108A) with ATc, and Rv0884c_cKDTet+Rv0884c with ATc; **e**, H37Rv) or treated with d-serine. Bacterial load in lung tissues at 4 weeks post infection was determined using c.f.u. assay. **g**, After co-culturing H37Rv-infected BMDMs (MOI = 5) and activated TB10Rg3 T cells treated with or without d-serine for 2 h, percentage of CD69^+^ TB10Rg3 T cells was measured in TB10Rg3 T cells. **h**, After co-culturing H37Rv-infected BMDMs (MOI = 5) and activated TB10Rg3 T cells treated with or without d-serine for 3 days, the expression of IFN-γ was measured using ELISA. **i**, After 1 day of infection with *Mtb* strains, BMDMs were co-incubated with activated TB10Rg3 T cells treated with or without d-serine for 3 days, and intracellular c.f.u. in BMDMs infected for 1 or 4 days were determined. No T, group of unpulsed BMDMs infected with H37Rv for 4 days without co-incubation with T cells; d1, group of unpulsed BMDMs infected with H37Rv for 1 day without co-incubation with T cells; d4, group of BMDMs infected with H37Rv for 4 days; Rg3, group of pulsed or unpulsed BMDMs infected with H37Rv for 4 days and co-incubated with TB10Rg3 T cells. **j**,**k**, C57BL/6 mice were aerosol-infected with ~200 c.f.u. per mouse of H37Rv and administered with d-serine for 4 weeks. Percentages of IFN-γ^+^ (**j**) and CD69^+^ (**k**) CD8^+^ T cells in lung tissues were measured; FMO was used as control. Data in **a**–**k** represent one experiment with at least three independent biological replicates. Results are shown as mean ± s.e.m. Two-way ANOVA with Tukey’s multiple comparisons test (**i**), one-way ANOVA with Tukey’s multiple comparisons test (**c**,**d**,**f**) and two-tailed unpaired Student’s *t*-test (**a**,**b**,**e**,**g**,**h**,**j**,**k**) were used for statistical analysis. Source data

*Mtb* mainly resists and impairs the myriad of macrophage defences^1,2^. To examine the functional role of d-serine in host innate immune responses to *Mtb* infection, the effect of d-serine on the intracellular survival of *Mtb* in macrophage was analysed by using the colony-forming unit (c.f.u.) assay. Addition of d-serine did not significantly change the intracellular survival of *Mtb* H37Rv in BMDMs (Fig. 3a left panel). Consistently, ATc-induced knockdown of Rv0884c did not significantly change the intracellular survival of *Mtb* H37Rv in BMDMs (Fig. 3a right panel). To directly analyse intracellular *Mtb* activity, LIVE/DEAD two-colour fluorescence assay of bacterial viability was performed. Similarly, ATc-induced knockdown of Rv0884c did not significantly affect the intracellular bacterial activity of *Mtb* H37Rv in BMDMs, indicated as the ratio of dead to live bacteria (Fig. 3b).

**Extended Data Fig. 2:**
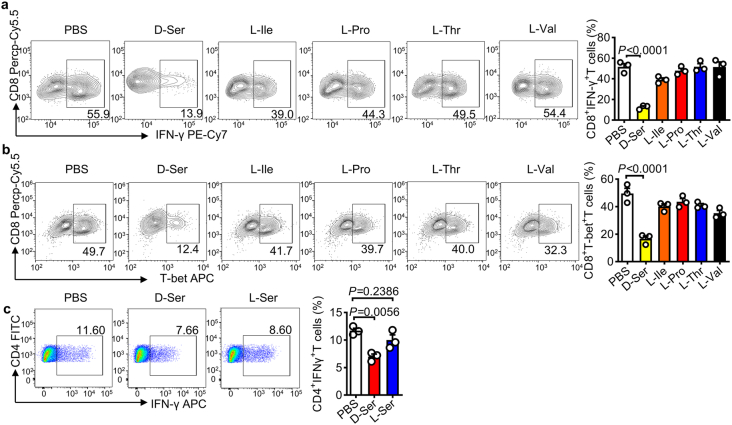
D-serine inhibits IFN-γ production by CD8^+^ and CD4^+^ T cells. (**a, b**) Percentages of CD8^+^ IFN-γ^+^ T cells (**a**) and CD8^+^ T-bet^+^ T cells (**b**) from differentiated CD8^+^ T cells treated with hypoxia-induced *Mtb* metabolites (10 mM) *in vitro* were analyzed by FACS. (**c**) Percentages of CD4^+^ IFN-γ^+^ T cells from differentiated Th1 cells treated with PBS, D-serine or L-serine (10 mM) were analyzed by FACS *in vitro*. All data represent one experiment with at least three independent biological replicates. Results are shown as mean ± s.e.m. One-way ANOVA with Tukey’s multiple comparisons test (**a-c**) was used for statistical analysis. Source data

CD8^+^ and CD4^+^ T cells promote the antimicrobial capacities of macrophages by secreting macrophage-activating cytokines, such as IFN-γ^13,30^. The role of d-serine in the differentiation of CD8^+^ or CD4^+^ T cells in vitro was previously investigated^31–34^. Treatment with d-serine (Extended Data Fig. 2a,b) significantly decreased the percentage of IFN-γ-secreted CD8^+^ T cells^32^ (Fig. 3c) and CD4^+^ T cells (Extended Data Fig. 2c). To test the hypothesis that the in vivo effects of d-serine were dependent on CD8^+^ T cells, we adoptively transferred CD8^+^ T cells from WT mice to *Rag1*^−/−^ mice (lacking T and B lymphocytes)^35^, and infected these animals with Rv0884c-knockdown or complemented strains. d-serine (30 g l^−1^) was administered via drinking water to half of the ATc-treated Rv0884c_cKD^Tet^-infected mice. The bacterial load in the lungs of the infected mice was measured after 4 weeks of infection. Treatment of d-serine or knockdown of Rv0884c had no significant impact on the bacterial load in the lung tissues of control *Rag1*^−/−^ mice, but knockdown of Rv0884c reduced, while addition of d-serine increased, the bacterial load in lung tissues of *Rag1*^−/−^ mice with transferred CD8^+^ T cells (Fig. 3d,e). The reduced survival of Rv0884c-knockdown *Mtb* was reversed by addition of d-serine (Fig. 3d). These results suggest that while d-serine has no direct effect on macrophage antimicrobial activity, it inhibits CD8^+^ T cell responses.

### d-serine inhibits IFN-γ production by CD8^+^ T cells

Next, we investigated whether d-serine impairs the activation of CD8^+^ T cells^36^. d-serine did not significantly change the activation of CD8^+^ T cells as indicated by CD69 expression on the surface of CD8^+^ T cells in vitro (Fig. 3f). Then, to confirm whether d-serine impairs the effector functions of CD8^+^ T cells^36^, we applied a T cell and macrophage co-culture ex vivo model. The TB10.4_4−11_-specific CD8^+^ T cell, referred to as TB10Rg3 T cells, was derived from knock-in mice expressing TB10Rg3 TCR, which specifically recognizes the *Mtb* TB10.4_4–11_ epitope^36^. It has been shown that TB10.4_4−11_-specific CD8^+^ T cells are unable to recognize *Mtb*-infected macrophages unless the macrophages are pulsed with the TB10.4_4–11_ peptide^36^. When *Mtb*-infected BMDMs were pulsed with the TB10.4_4−11_ peptide and then co-cultured with TB10Rg3 T cells treated with or not with d-serine, no differences in the percentage of CD69^+^ TB10Rg3 T cells were observed (Fig. 3g). However, with the same co-culture model, d-serine significantly inhibited IFN-γ release from TB10Rg3 T cells when co-cultured with TB10.4_4−11_ peptide-pulsed BMDMs (Fig. 3h). We also used the co-culture model to confirm whether d-serine indirectly reduced the ability of macrophages to restrict *Mtb* via inhibition of IFN-γ production by CD8^+^ T cells. d-serine-treated TB10Rg3 T cells were less able to restrict the intracellular growth of *Mtb* (Fig. 3i). Furthermore, upon *Mtb* H37Rv infection, d-serine-treated mice had a significantly lower percentage of IFN-γ-producing CD8^+^ T cells in their lung tissues than mice treated with phosphate buffered saline (PBS) (Fig. 3j). However, d-serine did not significantly change the activation of CD8^+^ T cells as indicated by the percentage of CD69^+^CD8^+^ T cells in the lung tissues of the infected mice (Fig. 3k).

### Rv0884c inhibits IFN-γ production in CD8^+^ T cells

**Fig. 4:**
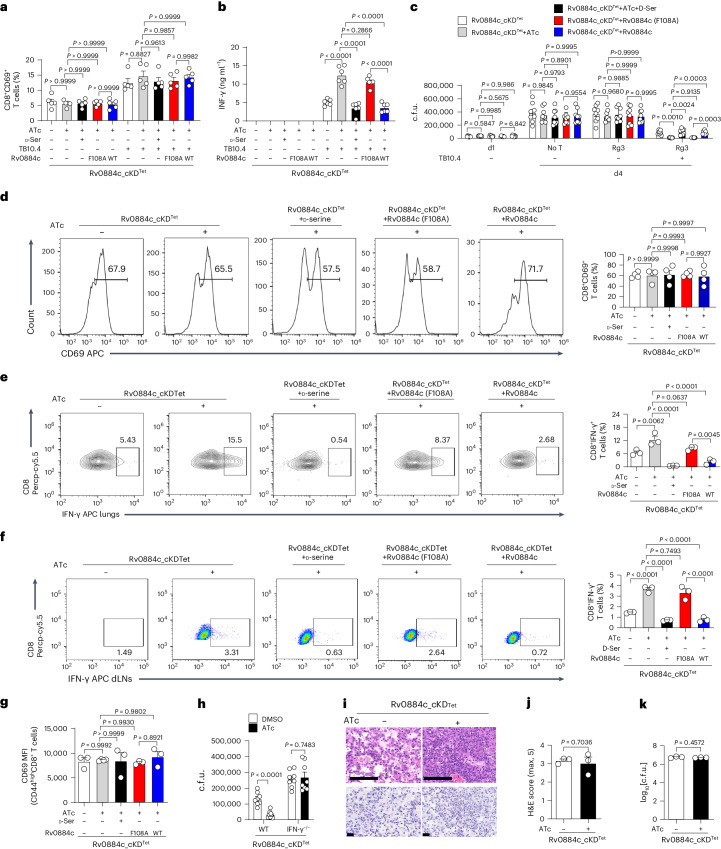
Rv0884c inhibits IFN-γ production by CD8^+^ T cells. **a**, After co-culturing indicated *Mtb* strains-infected BMDMs (MOI = 5) and activated TB10Rg3 T cells for 2 h, the percentage of CD69^+^ TB10Rg3 T cells was measured in TB10Rg3 T cells. **b**, After co-culturing indicated *Mtb* strains-infected BMDMs (MOI = 5) and activated TB10Rg3 T cells for 3 days, the expression of IFN-γ was measured using ELISA. **c**, After 1 day of infection with *Mtb* strains, BMDMs were co-incubated with activated TB10Rg3 T cells for 3 days and intracellular c.f.u. in BMDMs infected for 1 or 4 days were determined. **d**–**g**, C57BL/6 mice were aerosol-infected with ~200 c.f.u. per mouse of Rv0884c_cKD^Tet^ with or without ATc, Rv0884c_cKD^Tet^ with ATc and d-serine (30 g l^−1^), Rv0884c_cKD^Tet^+Rv0884c (F108A) with ATc, and Rv0884c_cKD^Tet^+Rv0884c with ATc. After 4 weeks of infection, we assayed: the percentage of CD69^+^CD8^+^ T cells (**d**); the percentage of CD8^+^IFN-γ^+^ T cells in CD8^+^ T cells from lung tissues (**e**), or from dLNs (**f**); and CD69 MFI of CD44^high^ CD8^+^ T cells in CD8^+^ T cells from lung tissues (**g**). **h**, Peptide (TB10.4_4–11_)-pulsed BMDMs were infected with Rv0884c_cKD^Tet^ treated with or without ATc for 1 day and then co-cultured with in vitro differentiated CD8^+^ T cells from spleens of C57BL6 mice or CD4^Cre^*Ifng*^fl/fl^ mice for another 3 days. Bacterial burden was measured 4 days after infection. **i**–**k**, CD4^Cre^*Ifng*^*fl/fl*^ mice were aerosol-infected with ~200 c.f.u. per mouse of Rv0884c_cKD^Tet^ with or without ATc for 4 weeks. Histopathology of lung sections from infected mice was assessed via H&E staining (**i**, top; scale bar, 100 μm), acid-fast staining (**i**, bottom; scale bar, 20 μm), histology score (**j**) and bacterial load (**k**). Data in **a**–**k** represent one experiment with at least three independent biological replicates. Results are shown as mean ± s.e.m. Two-way ANOVA with Tukey’s multiple comparisons test (**c**), one-way ANOVA with Tukey’s multiple comparisons test (**a**,**b**,**d**–**g**) and two-tailed unpaired Student’s *t*-test (**h**,**j**,**k**) were used for statistical analysis. Source data

To show that d-serine produced by *Mtb* via Rv0884c during infection could have the same effect as exogenously applied d-serine, we performed experiments with Rv0884c-knockdown or complemented strains as above. Confirming that activation of CD8^+^ T cells was not affected by d-serine or Rv0884c activity, we observed that the percentage of CD69^+^ TB10Rg3 T cells was not significantly changed when co-cultured with BMDMs infected with Rv0884c-knockdown or its complemented strains (Fig. 4a). However, decreased concentrations of IFN-γ were detected in TB10Rg3 T cells co-cultured with BMDMs infected with Rv0884c-expressing *Mtb* H37Rv, compared with those co-cultured with BMDMs infected with either Rv0884c-knockdown or Rv0884c (F108A)-complemented *Mtb* strains (Fig. 4b). Intramacrophage growth of the knockdown- or mutant-complemented strains was also reduced in these co-culture experiments (Fig. 4c). d-serine treatment was able to rescue both phenotypes in Rv0884c-knockdown infection conditions (Fig. 4b,c).

**Extended Data Fig. 3:**
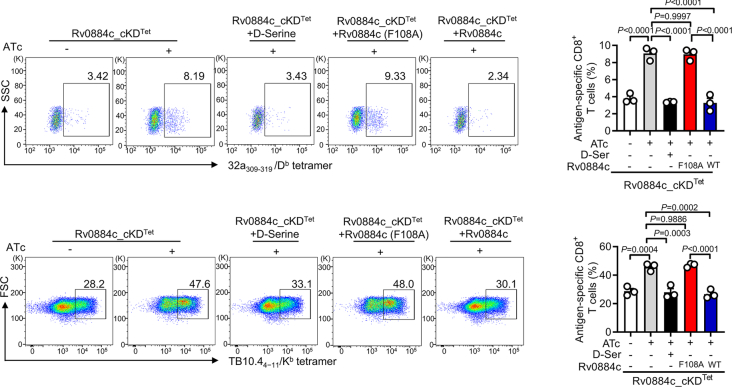
Effect of Rv0884c/D-serine on the percentage of *Mtb* antigen-specific CD8 T cells in lung tissues. C57BL/6 mice were aerosol-infected with approximately 200 c.f.u. per mouse of Rv0884c_cKD^Tet^ with or without ATc, Rv0884c_cKD^Tet^ with ATc and D-serine (30 g/L), Rv0884c_cKD^Tet^+Rv0884c (F108A) with ATc, Rv0884c_cKD^Tet^+Rv0884c with ATc. At 4 weeks post infection, percentage of 32a_309-319_ specific and TB10.4_4−11_ specific CD8^+^ T cells from CD8^+^ T cells in lung tissues was detected by FACS. All data represent one experiment with at least three independent biological replicates. Results are shown as mean ± s.e.m. One-way ANOVA with Tukey’s multiple comparisons test was used for statistical analysis. Source data

**Extended Data Fig. 4:**
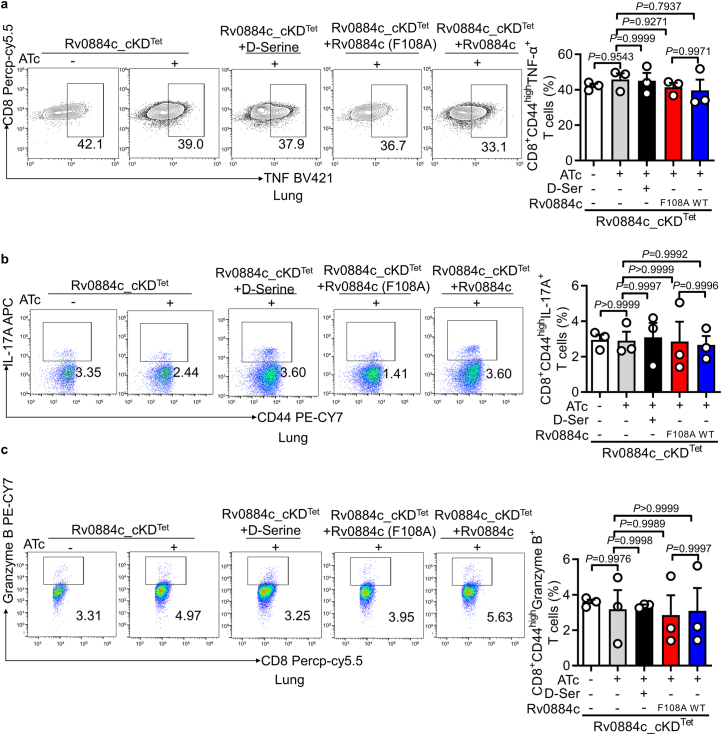
Effect of Rv0884c on the different subsets of CD8 T cells in lung tissues. (**a-c**) C57BL/6 mice were aerosol-infected with approximately 200 c.f.u. per mouse of Rv0884c_cKD^Tet^ with or without ATc, Rv0884c_cKD^Tet^ with ATc and D-serine (30 g/L), Rv0884c_cKD^Tet^+Rv0884c (F108A) with ATc, Rv0884c_cKD^Tet^+Rv0884c with ATc. At 4 weeks post infection, we assayed: percentage of CD8^+^CD44^high^TNF-α^+^ T cells (**a**), CD8^+^CD44^high^IL-17A^+^ T cells (**b**), and CD8^+^CD44^high^granzyme B^+^ T cells (**c**) from the CD8^+^CD44^high^ T cells in lung tissues by FACS. All data represent one experiment with at least three independent biological replicates. Results are shown as mean ± s.e.m. One-way ANOVA with Tukey’s multiple comparisons test (**a-c**) was used for statistical analysis. Source data

Analysing these phenotypes during in vivo infection, knockdown or mutation of Rv0884c did not significantly change the percentage of CD69^+^CD8^+^ T cells in the lung tissues of the infected mice (Fig. 4d). However, lung tissues and dLNs of mice infected with Rv0884c-knockdown strains had a significantly higher percentage of IFN-γ-producing CD8^+^ T cells than those of mice infected with Rv0884c-expressing bacteria (Fig. 4e,f left panels 1 and 2). This phenotype was rescued by complementation of Rv0884c, but not Rv0884c (F108A) (Fig. 4e,f left panels 2, 4 and 5). CD44 expression is an indicative marker for effector T cells^37,38^. To exclude the effect of Rv0884c and d-serine on the activation of effector CD8^+^ T cells, we monitored the CD69 expression on CD44^high^ CD8^+^ T cells. Knockdown or mutation of Rv0884c did not significantly change the levels of CD69 expression on CD44^high^ CD8^+^ T cells (Fig. 4g). Then, we investigated whether Rv0884c impairs the effector functions of *Mtb*-specific CD8^+^ T cells through d-serine^36^. Treatment with d-serine reversed the increase in percentage of IFN-γ-producing, 32a_309–319_- or TB10.4_4−11_-specific CD8^+^ T cells in the lung tissues of control mice infected with the Rv0884c-knockdown *Mtb* strain^36,39^ (Fig. 4e left panels 2 and 3, and Extended Data Fig. 3), but did not significantly change the level of TNF-α, IL-17 or granzyme B of CD44^high^ CD8^+^ T cells from the lung tissues of *Mtb*-infected mice (Extended Data Fig. 4a–c). To confirm the involvement of IFN-γ in the CD8^+^ T cell-dependent restriction of intramacrophage *Mtb*, we analysed bacterial burden in BMDMs and Cd4^Cre^*Ifng*^*fl/fl*^ mice. BMDMs co-cultured with CD8^+^ T cells derived from Cd4^Cre^*Ifng*^*fl/fl*^ mice did not show significantly lower intracellular burden when infected with Rv0884c-knockdown strains than with WT strains (Fig. 4h). Consistently, knockdown of Rv0884c did not reduce the bacterial burden or pathology in the lung tissues of the infected Cd4^Cre^*Ifng*^*fl/fl*^ mice compared with that of mice infected with control *Mtb*, while it did in WT mice (Figs. 2c–f and 4i–k). These results indicate that Rv0884c may suppress host immunity by downregulating CD8^+^ T cell-derived IFN-γ.

### Rv0884c-derived d-serine inactivates mTORC1 in CD8^+^ T cells

**Fig. 5:**
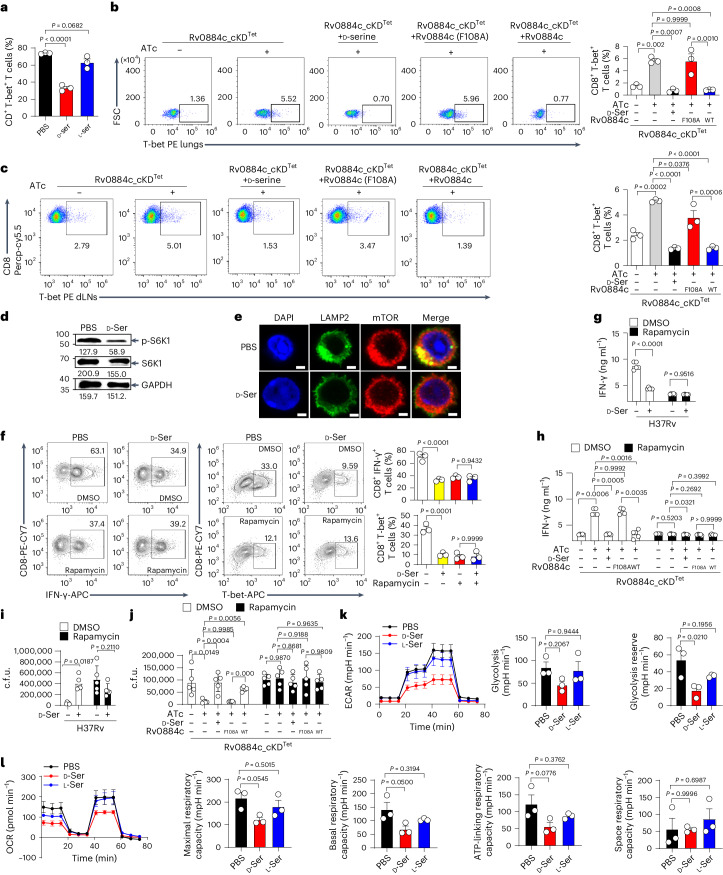
Rv0884c/d-serine inhibits IFN-γ production by CD8^+^ T cells via inactivation of mTORC1. **a**,**d**,**e**, Naïve CD8^+^ T cells were stimulated with anti-CD3 antibodies, anti-CD28 antibodies, IL-2 and IL-12 p70 for 5 days and treated with PBS, d-serine or l-serine. Percentage of T-bet^+^ CD8^+^ T cells was analysed using fluorescence-activated cell sorting (FACS) (**a**); phosphorylation of S6K1 was assayed (**d**) and levels of S6K1, p-S6K1 and GAPDH were quantified using ImageJ and are indicated below blots (**d**); co-localization of lysosomes with mTOR was analysed (**e**; scale bar, 2 μm). **b**,**c**, C57BL/6 mice were aerosol-infected with indicated *Mtb* strains. Expression of T-bet in CD8^+^ T cells from lung tissues (**b**) and dLNs (**c**) was analysed using FACS. **f**, Naïve CD8^+^ T cells were stimulated with anti-CD3 antibodies, anti-CD28 antibodies, IL-2 and IL-12 p70 for 5 days and treated with PBS, d-serine or mTORC1 inhibitor rapamycin. Expression of IFN-γ (left group) and T-bet (middle group) was assayed via FACS and quantified as percentage of CD8^+^ T cells (right). **g**,**h**, BMDMs infected with H37Rv (**g**) or indicated *Mtb* strains (**h**) for 1 day were co-incubated with TB10Rg3 T cells treated with or without d-serine or rapamycin for another 3 days. The expression of IFN-γ was measured using ELISA. **i**,**j**, BMDMs infected with H37Rv (**i**) or indicated *Mtb* strains (**j**) for 1 day were co-incubated with TB10Rg3 T cells treated with or without d-serine or rapamycin for another 3 days. Intracellular c.f.u. in BMDMs were determined. **k**,**l**, Naïve CD8^+^ T cells were stimulated with anti-CD3 and anti-CD28 antibodies in the presence of IL-2 for 24 h and treated with PBS, d-serine or l-serine. ECAR (**k**) and OCR (**l**) were measured and analysed via Seahorse metabolic assay. Data in **a**–**l** represent one experiment with at least three independent biological replicates. Results are shown as mean ± s.e.m. Two-way ANOVA with Tukey’s multiple comparisons test (**g**–**j**) and one-way ANOVA with Tukey’s multiple comparisons test (**a**–**c**,**f**,**k**,**l**) were used for statistical analysis. Source data

**Extended Data Fig. 5:**
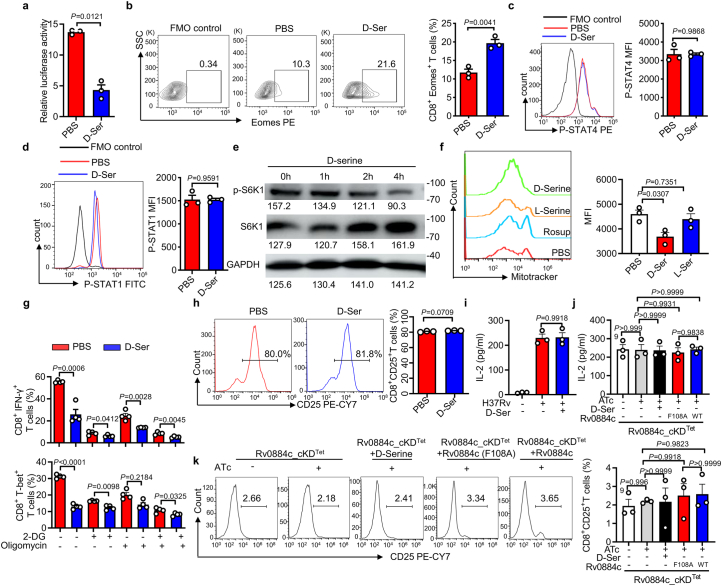
Rv0884c/D-serine inhibits the IFN-γ production of CD8^+^ T cells by inactivating mTORC1. (**a**) Expression of T-bet reporter gene treated with PBS or D-serine (10 mM) was detected using dual luciferase reporter gene assay. (**b-d**) Expression of p-STAT1 (**d**), p-STAT-4 (**c**) or Eomes (**b**) in CD8^+^ T cells treated with PBS or D-serine (10 mM) were analyzed by FACS *in vitro*. (**e**) Phosphorylation of S6K1 in 293 T cells treated with PBS or D-serine (10 mM) was measured using western blot. Values below blots indicate densitometry quantification with ImageJ. (**f**) Mitochondrial mass was measured in primary CD8^+^ T cells treated with PBS, L-serine or D-serine (10 mM). (**g**) Naïve CD8^+^ T cells were stimulated with anti-CD3 in the presence of anti-CD28 antibodies, IL-2 and IL-12 p70 for 5 days and treated with PBS, D-serine (10 mM), 2-DG (4 mM) and Oligomycin (1 μM). Expression of IFN-γ (up) and T-bet (down) from the CD8^+^ T cells was assayed. (**h**) Naïve CD8^+^ T cells were stimulated with anti-CD3 in the presence of anti-CD28 antibodies, IL-2 and IL-12 p70 for 5 days and treated with PBS or D-serine (10 mM). Percentage of CD25^+^CD8^+^ T cells from the CD8^+^ T cells was detected by FACS. (**i**) The IL-2 level of serum from H37Rv-infected mice was detected by ELISA. (**j, k**) C57BL/6 mice were aerosol-infected with indicated *Mtb* strains. At 4 weeks post infection, IL-2 level of serum was detected by ELISA (**j**); Percentage of CD25^+^CD8^+^ T cells from the CD8^+^ T cells in lung tissues was detected by FACS (**k**). All data represent one experiment with at least three independent biological replicates. Results are shown as mean ± s.e.m. One-way ANOVA with Tukey’s multiple comparisons test (**f, i-k**) and Two-tailed unpaired Student’s t-test (**a-d, g-h**) were used for statistical analysis. Source data

IFN-γ transcription by CD8^+^ T cells is regulated by two key transcription factors, T-bet and Eomes^40^. To explore the mechanism by which d-serine affects IFN-γ expression, we tested whether d-serine inhibited the activity of either of these transcription factors. d-serine significantly inhibited the luciferase activity of the T-bet-luc reporter gene in HEK293T cells (Extended Data Fig. 5a). Consistently, treatment with d-serine significantly inhibited the expression of T-bet in CD8^+^ T cells in vitro (Fig. 5a), but increased the expression of Eomes (Extended Data Fig. 5b). In vivo, mice infected with Rv0884c-knockdown *Mtb* had significantly higher percentages of T-bet^+^ CD8^+^ T cells in the lung tissues (Fig. 5b left panels 1 and 2) or dLNs (Fig. 5c left panels 1 and 2) compared with mice infected with control Rv0884c-expressing *Mtb*. Conversely, treatment with d-serine eliminated the increase in T-bet^+^ CD8^+^ T cells observed in the lung tissues or dLNs of mice infected with Rv0884c-knockdown *Mtb* (Fig. 5b,c left panels 2 and 3). The increase in T-bet^+^ CD8^+^ T cells upon infection with the Rv0884c-knockdown strain was rescued when the strain was complemented with Rv0884c, but not with the Rv0884c (F108A)-mutant construct (Fig. 5b,c left panels 2, 4 and 5).

T-bet is induced via an upstream signal transducer and activator of transcription 1 (STAT-1), STAT-4 and mTOR signalling pathway in CD8^+^ T cells^40–45^. Treatment with d-serine did not significantly change phosphorylation of STAT-1 or STAT-4 in CD8^+^ T cells in vitro (Extended Data Fig. 5c,d), but markedly reduced activation of mTORC1. This was shown by decreased phosphorylation at site 371 of S6K1, a protein downstream of mTORC1 (Fig. 5d and Extended Data Fig. 5e), and decreased lysosome-associated mTOR in CD8^+^ T cells (Fig. 5e). Consistently, in an in vitro differentiation model, treatment of T cells with rapamycin, an inhibitor of mTORC1, significantly inhibited the percentage of both CD8^+^IFN-γ^+^ T cells and T-bet^+^ CD8^+^ T cells (Fig. 5f left panels of left and middle groups). Using the same model, blockade of mTORC1 by rapamycin also eliminated the inhibitory effect of d-serine on the expression of T-bet and IFN-γ (Fig. 5f right panels of left and middle groups) and upon expression of IFN-γ by TB10Rg3 T cells in a d-serine-treated BMDMs-TB10Rg3 T cells co-culture model (Fig. 5g). Using the same model, we found that knockdown of Rv0884c was no longer able to increase the secretion of IFN-γ from rapamycin-treated TB10Rg3 T cells (Fig. 5h). Furthermore, treatment of TB10Rg3 T cells with rapamycin eliminated the effects of d-serine treatment on the intracellular survival of *Mtb* in peptide (TB10.4_4–11_)-pulsed, H37Rv-infected BMDMs (Fig. 5i), and abrogated the Rv0884c-dependent increase in intracellular survival of *Mtb* (Fig. 5j).

Since mTORC1 is a cellular energy receptor that is closely associated with mitochondrial function^46,47^, the effect of d-serine on glycolysis and oxidative phosphorylation of mitochondria was measured. Using Seahorse XF Analyzer, we found that d-serine significantly inhibited glycolysis (ECAR) and oxidative phosphorylation (OCR) in primary CD8^+^ T cells (Fig. 5k,l) and simultaneously significantly reduced mitochondrial mass detected in CD8^+^ T cells (Extended Data Fig. 5f). Inhibition of glycolysis by 2-deoxy-d-glucose (2-DG)^48^, or oxidative phosphorylation by oligomycin^49,50^, in CD8^+^ T cells decreased the percentage of CD8^+^IFN-γ^+^ T cells or T-bet^+^ CD8^+^ T cells, but did not eliminate the inhibitory effect of d-serine on CD8^+^ T cell differentiation (Extended Data Fig. 5g). We also examined whether d-serine and Rv0884c affect IL-2R signalling, which is known to induce mTORC1 activation to support transcriptional programmes involved in the optimal differentiation of effector CD8^+^ T cells^43^. d-serine had no significant effect on the percentage of CD8^+^ T cells expressing CD25, the α chain of IL-2 receptor^43^, in vitro (Extended Data Fig. 5h). Furthermore, d-serine and Rv0884c affected neither the serum IL-2 levels in *Mtb* H37Rv-infected mice (Extended Data Fig. 5i,j) nor the percentage of CD25^+^ CD8^+^ T cells in the lung tissues of *Mtb* H37Rv-infected mice (Extended Data Fig. 5k). These results suggest that d-serine may inhibit the activation of mTORC1 in CD8^+^ T cells.

### d-serine interacts with WDR24

**Fig. 6:**
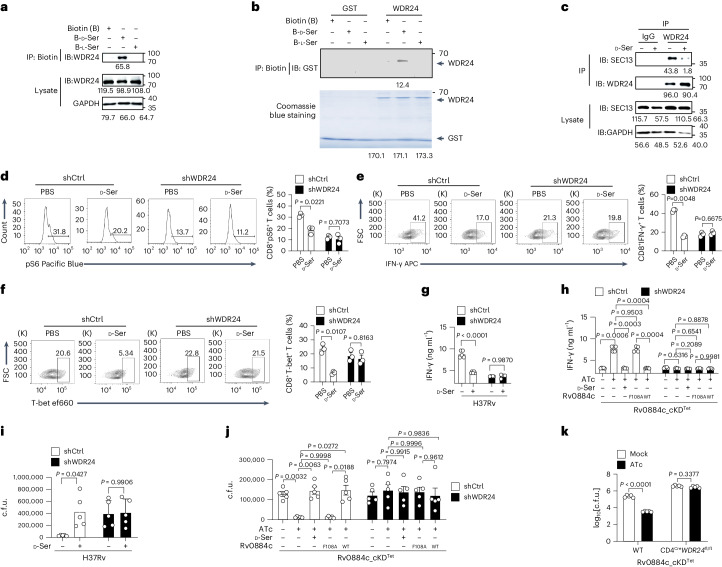
d-serine interacts with WDR24 to suppress anti-TB immunity functions through IFN-γ. **a**, Co-IP was performed to detect the interaction between d-serine and WDR24 in T cells. Values below blots indicate densitometry quantification with ImageJ. **b**, Direct interaction of d-serine with WDR24 was detected using GST pull-down assay in vitro. Values below blots indicate densitometry quantification with ImageJ. **c**, Co-IP was performed to detect the interaction of WDR24 with other components of GATOR2 (SEC13), which is interrupted by d-serine. Values below blots indicate densitometry quantification with ImageJ. **d**–**f**, *WDR24*-knockdown CD8^+^ T cells were treated with PBS or d-serine. The phosphorylation of S6 (**d**), the expression of T-bet (**e**) and IFN-γ (**f**) in WT CD8^+^ T cells or *WDR24*-knockdown CD8^+^ T cells were analysed using FACS. **g**,**i**, BMDMs infected with H37Rv (MOI = 5) for 1 day were co-incubated with TB10Rg3 T cells or *WDR24*-knockdown TB10Rg3 T cells treated with or without d-serine (10 mM) for another 3 days. The expression of IFN-γ was measured using ELISA (**g**); intracellular c.f.u. in BMDMs were determined (**i**). **h**,**j**, BMDMs were infected with indicated *Mtb* strains (MOI = 5) for 1 day and co-incubated with TB10Rg3 T cells or *WDR24-*knockdown TB10Rg3 T cells for another 3 days. The expression of IFN-γ was measured using ELISA (**h**); intracellular c.f.u. in BMDMs were determined (**j**). **k**, CD4^Cre^*WDR24*^*fl/fl*^ mice were aerosol-infected with ~200 c.f.u. per mouse of Rv0884c_cKD^Tet^ treated with or without ATc for 4 weeks. Bacterial load in lung tissues was determined using c.f.u. assay. Data in **a**–**k** represent one experiment with at least three independent biological replicates. Results are shown as mean ± s.e.m. Two-way ANOVA with Tukey’s multiple comparisons test (**d**–**k**) was used for statistical analysis. Source data

**Extended Data Fig. 6:**
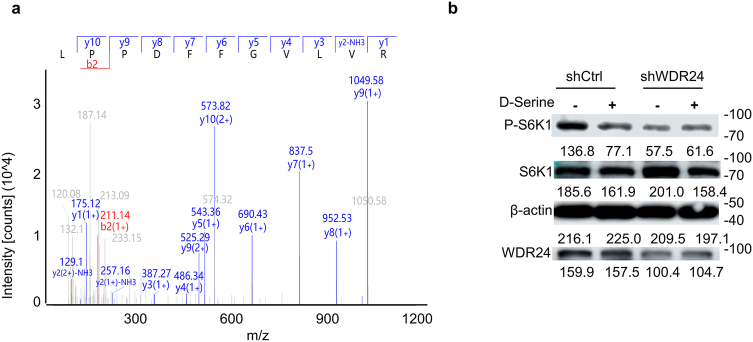
D-serine inhibits mTORC1 activity via WDR24. (**a**) Representative mass spectrum of interacting proteins for biotin-D-serine identified by LC-MS/MS analysis. (**b**) WT and *WDR24* knockdown 293 T cells were treated with PBS or D-serine (10 mM). Phosphorylation of S6K1 was assayed. Levels of S6K1, p-S6K1, β-actin and WDR24 were quantified with ImageJ. Values below blots indicate densitometry quantification with ImageJ. Data of **b** represents one experiment with at least three independent biological replicates. Source data

To further characterize the pathway through which Rv0884c-derived d-serine inactivates mTORC1 in CD8^+^ T cells, biotin-labelled d-serine was synthesized and co-immunoprecipitated with the cell lysate of Jurkat cells. Immunoprecipitation mass spectrometry (IP–MS) analysis of the proteins co-immunoprecipitating with d-serine was performed and WDR24 was identified as a d-serine-interacting protein (Extended Data Fig. 6a). WDR24 is a subunit of the GAP activity towards Rags 2 (GATOR2) complex in T cells, which is an activator of the metabolic regulator mTORC1. The interaction was further confirmed by co-IP in CD8^+^ T cells (Fig. 6a). Glutathione S-transferase (GST) pull-down assay demonstrated that d-serine directly interacted with WDR24 (Fig. 6b). As expected, WDR24 interacted with SEC13, another subunit of GATOR2 complex under endogenous conditions to form the GATOR2 complex^51^ in CD8^+^ T cells (Fig. 6c). However, d-serine (10 mM) treatment abrogated the interaction of WDR24 with SEC13 (Fig. 6c). To determine whether d-serine inhibits the activation of mTORC1 in CD8^+^ T cells through WDR24, expression of WDR24 was knocked down by specific short hairpin RNA (shRNA) in CD8^+^ T cells. Knockdown of WDR24 eliminated the inhibitory effects of d-serine (10 mM) on S6 phosphorylation (Fig. 6d and Extended Data Fig. 6b) and on expression of T-bet and IFN-γ (Fig. 6e,f). Likewise, WDR24-knockdown TB10Rg3 T cells treated with d-serine (10 mM) did not show the reduced production of IFN-γ when co-cultured with peptide (TB10.4_4–11_)-pulsed, *Mtb* H37Rv-infected BMDMs (Fig. 6g). Using the same model, we found that knockdown of WDR24 in TB10Rg3 T cells eliminated the inhibitory effect of Rv0884c on the secretion of IFN-γ (Fig. 6h). Furthermore, treatment of WDR24-knockdown TB10Rg3 T cells with d-serine (10 mM) failed to promote intracellular survival of *Mtb* in peptide (TB10.4_4–11_)-pulsed BMDMs (Fig. 6i). Likewise, the enhanced effect of Rv0884c on the intracellular survival of *Mtb* in BMDMs was not observed when WDR24 was knocked down in TB10Rg3 T cells (Fig. 6j). WT or CD4^Cre^*WDR24*^*fl/fl*^ mice fed with or without ATc were infected with the Rv0884c_cKD^Tet^ strain. The reduction of the bacterial burden in the WT mice fed with ATc was not observed in the CD4^Cre^*WDR24*^*fl/fl*^ mice (Fig. 6k). These results indicate that d-serine may directly interact with WDR24 to disrupt the formation of GATOR2 complex, thus inhibiting mTORC1 activity and CD8^+^ T cell-dependent restriction of intramacrophage *Mtb*.

## Discussion

As an intracellular bacterium, *Mtb* must adapt to environmental conditions such as hypoxia to establish successful persistent infection inside macrophages or granulomas. We discovered that hypoxia induces d-serine production by *Mtb*, which inhibits mTORC1 and T-bet induction to restrict downstream CD8^+^ T cell immune responses, macrophage activation and control of *Mtb* infection in mice. It has been shown that hypoxic granulomas are formed in lung tissues from guinea pigs and non-human primates, but not in C57BL/6 mice infected with *Mtb*^3,27^. Recent studies show that C3HeB/FeJ mice are a better model for studying hypoxia and immunopathology during *Mtb* infection. Although C57BL/6 mice infected with *Mtb* have been shown to have slightly lowered pO_2_ (∼37 mm Hg), the animals fail to produce highly organized caseous or necrotic lesions, or develop hypoxic regions within their infected lungs^17,52^. Despite these limitations, the intracellular oxygen tensions in macrophages infected with *Mtb* have been functionally approximated to be 1%, similar to hypoxic conditions, despite incubation under ambient tensions of 10% or even 21% (ref. ^7^). Accordingly, our pimonidazole staining suggest that both the lung tissues and dLNs of the *Mtb*-infected C57BL/6 mice exhibited a hypoxic state, compared with that in uninfected mice or mice exposed to 95% O_2_. Therefore, although C57BL/6 mice infected with *Mtb* may not be the best model for analysing hypoxic granulomas, particularly compared with C3HeB/FeJ mice^53^, hypoxic conditions can develop in the lungs and dLNs. In future work, we will use C3HeB/FeJ mice to further explore the function of hypoxia-induced Rv0884c-derived d-serine in the development of tuberculous granuloma.

Following standard GC–MS metabolic profiling procedures^54–58^ and bacterial genetics, we found that under hypoxia, *Mtb* produced high levels of d-serine through the phosphoserine aminotransferase Rv0884c and dependent on its aminotransferase active site residue, F108. *Mtb* depends on phosphoglycerate dehydrogenase (SerA) to catalyse the oxidation of d-3-phosphoglycerate to 3-phosphohydroxypyruvate^29^, phosphoserine aminotransferase (SerC, Rv0884c) to convert phosphohydroxypyruvate to l-3-phosphoserine^29^ and phosphoserine phosphatase (SerB) to dephosphorylate l-3-phosphoserine to l-serine^59^. However, d-serine is derived from transposition of l-serine, but it remains unknown which enzyme catalyses the formation of d-serine from l-serine in *Mycobacteria*^59^. Therefore, additional enzymes may be involved in converting l-serine to d-serine during *Mtb* infection under hypoxia, which needs further investigation.

Basal levels of Rv0884c or d-serine are essential for the aerobic growth of pathogenic *Mycobacteria* in vitro^25,26,28^. It is possible that this might have contributed to in vivo phenotypes. However, our present data demonstrate that Rv0884c or d-serine inhibits the growth of *Mycobacteria* under anaerobic conditions in vitro. Furthermore, Rv0884c or d-serine did not significantly affect the intracellular survival and bacterial viability of *Mtb* H37Rv in macrophages ex vivo but promoted the survival of *Mycobacteria* in hypoxic conditions in vivo. Conditional knockdown of Rv0884c did not significantly change the bacterial load in the lung tissues of *Mtb*-infected *Rag1*^−/−^ mice but reduced the survival of *Mtb* in *Rag1*^−/−^ mice upon CD8^+^ T cell transfer. Therefore, in the context of host-associated hypoxia, Rv0884c does not directly affect the fitness of *Mtb*. However, by increasing d-serine levels, it indirectly facilitates *Mtb* immune evasion via inhibition of T cell and macrophage responses. We could not exclude the possibility that Rv0884c-derived d-serine may hijack additional host factors, which warrants further investigation.

Our data demonstrate that an *Mtb*-derived metabolite, d-serine, markedly inhibits the differentiation of CD8^+^ T cells into IFN-γ-secreted cells^60^ and inhibits their subsequent responses in the lung tissues of H37Rv-infected mice. We have also observed the inhibitory effect of d-serine on the generation of IFN-γ^+^ CD4^+^ T cells ex vivo. Future research is needed to analyse whether mycobacterial metabolites inhibit other adaptive immune cells and whether relieving any inhibition could increase the efficacy of TB vaccines.

mTOR signalling is crucial for mediating T cell activation and differentiation: CD8^+^ T cells with deficient mTORC1 activity are hindered in their differentiation into effector cells^44,45^. We found that treatment of the CD8^+^ T cells with rapamycin, a small-molecule inhibitor of the protein kinase mTOR^61,62^, eliminated the inhibitory effect of d-serine/Rv0884c on the expression of T-bet and IFN-γ in CD8^+^ T cells and also the expression of IFN-γ in TB10Rg3 T cells. This suggests that d-serine and Rv0884c activity may inhibit the expression of T-bet and IFN-γ by blocking the activation of mTORC1 in CD8^+^ T cells. Unlike the sensing of other amino acids (such as leucine by SAR1B, and sestrin 2 or arginine by GATOR1) that activates the mTORC1 pathway^63–66^, direct interaction of d-serine with GATOR2 inhibits mTORC1 activation and thus reduces T-bet-dependent secretion of IFN-γ. Our discovery of GATOR2 disruption by d-serine provides a mechanism for the inactivation of mTORC1 by amino acids that circumvents the sensing by different GATOR2 modulators.

**Extended Data Fig. 7:**
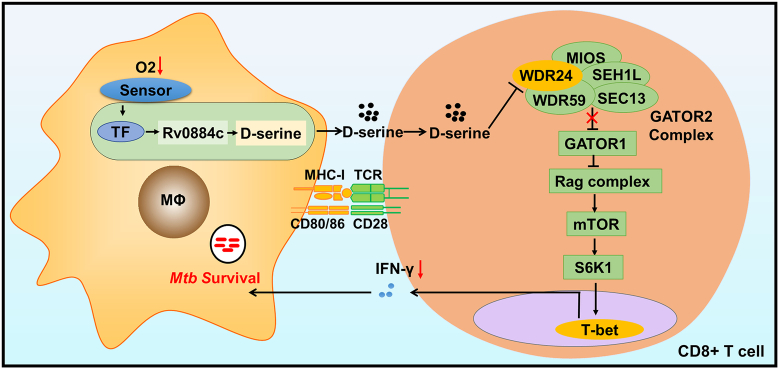
Summary diagram. Hypoxia conditions induce *Mtb* D-serine production via promoting the expression of phosphoserine aminotransferase Rv0884c. In CD8^+^ T cells, D-serine directly interacts with WDR24, a subunit of GATOR2, to inhibit mTORC1 mediated T-bet expression and IFN-γ secretion, leading to *Mtb* survival (The black arrows represent ‘induce’ or ‘promote’; The red downward arrow indicates a decrease in expression of IFN-γ; The red cross on the T cell side represent ‘inhibit’).

Collectively, our findings provide evidence that *Mycobacteria* have evolved to integrate adaptation to hypoxia with the suppression of CD8^+^ cell responses by enhancing biosynthesis of d-serine, thus facilitating the survival of *Mycobacteria* in the hypoxic and immune-hostile environment of macrophages (Extended Data Fig. 7).

## Methods

### Ethics consideration

All animal experiments were reviewed and approved by the Animal Experiment Administration Committee of Tongji University School of Medicine (TJAA06520101), and conducted in accordance with the National Institutes of Health (NIH) Guidelines for the Care and Use of Laboratory Animals.

### Bacterial culture

Bacterial strains used in this study are described in Supplementary Table 4. *Mtb* strains were cultured in Middlebrook 7H9 broth (BD) supplemented with 10% oleic acid–albumin–dextrose–catalase (OADC) and 0.05% Tween-80 (Sigma) or Middlebrook 7H10 agar (BD) supplemented with 10% OADC and antibiotics as required. The antibiotics used were 50 μg ml^−1^ of kanamycin, and 100 or 200 ng ml^−1^ of anhydrotetracycline (ATc). *Mtb* strains were grown to mid-log phase (optical density at 600 nm (OD_600_) ~0.6). The colony-forming unit (c.f.u.) ml^−1^ was measured by plating serial dilutions on 7H10 agar and counting the colonies after 4 weeks of incubation at 37 °C. Aliquots of bacterial cultures were prepared in 20% glycerol and Middlebrook 7H9 medium, and stored at −80 °C for subsequent use in infection of macrophages and mice, western blotting, quantitative polymerase chain reaction (qPCR) detection, or d-serine and l-serine quantitation assay. The viability of the bacterial suspensions was determined by staining, using the LIVE/DEAD BacLight bacterial viability kit (Thermo Fisher, L7012)^67^.

### Cell culture

HEK293T cells (ATCC CRL-3216) and iBMDMs^68^ (provided by F. Shao, National Institute of Biological Sciences, Beijing) were maintained in Dulbecco’s modified Eagle’s medium (DMEM, Gibco) supplemented with 10% (v/v) FBS and 1% (v/v) penicillin–streptomycin. The Jurkat cell line (ATCC TIB-152) and BMDMs derived from bone marrow cells in vitro in the presence of macrophage colony-stimulating factor (M-CSF) (Abclone, RP01221) at 20 ng ml^−1^ were cultured in RPMI 1640 (Gibco) supplemented with 10% (v/v) FBS and 1% (v/v) penicillin–streptomycin. All cells were routinely tested for contamination by mycoplasma.

### Plasmids, reagents and antibodies

Plasmids used in this study are described in Supplementary Table 4. The following reagents and antibodies were used for western blotting or immunoprecipitation: anti-SigA (BioLegend, 663205; 1:1,000 dilution in immunoblotting), anti-Rv0884c (ABclonal, 1:1,000 dilution in immunoblotting), anti-p-S6K1 (CST, 9234; 1:1,000 dilution in immunoblotting), anti-S6K1 (CST, 9202; 1:1,000 dilution in immunoblotting), anti-mTOR (CST, 2983; 1:100 dilution in immunostaining), anti-GAPDH (Abcam, 128915; 1:10,000 dilution in immunoblotting), anti-Sec13 (Santa Cruz, 514308; 1:1,000 dilution in immunoblotting and immunoprecipitation), anti-WDR24 (Proteintech, 20778-1-AP; 1:1,000 dilution in immunoblotting; 1:50 dilution in immunoprecipitation), anti-β-actin (Sigma, A5441; 1:5,000 dilution in immunoblotting and immunoprecipitation), anti-GAPDH (Sigma, G9545; 1:5,000 dilution in immunoblotting and immunoprecipitation), goat anti-rabbit IgG antibody, peroxidase conjugated (Sigma, AP132P, 1:5,000 dilution in immunoblotting and immunoprecipitation), streptavidin magnetic beads (MCE, HY-K0208) and protein A/G magnetic beads (MCE, HY-K0202). The following reagents and antibodies were used for immunostaining: anti-LAMP2 (Thermo Fisher, MA1-205; 1:100 dilution in immunostaining), DAPI (Thermo Fisher, 62248), donkey anti-mouse IgG (H + L) highly cross-adsorbed secondary antibody, Alexa Fluor 488 (Thermo Fisher, A-21202; 1:100 dilution in immunostaining), goat anti-rabbit IgG (H + L) cross-adsorbed secondary antibody, Alexa Fluor 568 (Thermo Fisher, A-11011; 1:100 dilution in immunostaining), goat anti-mouse IgG (H + L) highly cross-adsorbed secondary antibody, Alexa Fluor 568 (Thermo Fisher, A-11031; 1:100 dilution in immunostaining) and goat anti-rabbit IgG (H + L) highly cross-adsorbed secondary antibody, Alexa Fluor 488 (Thermo Fisher, A-11034; 1:100 dilution in immunostaining). The following reagents and antibodies were used for flow cytometry: anti-IFNγ (Biolegend, XMG1.2; 1:200), anti-P-STAT-4(Tyr693) (Invitrogen, 4LURPIE; 1:200), anti-CD3e (eBioscience, 145-2C11; 1:200), anti-CD4 (Biolegend, GK1.5; 1:200), anti-P-STAT-1(Ser727) (Invitrogen, Stat1S727-C6; 1:200), anti-CD44 (Invitrogen, IM7; 1:200), anti-EOMES (Invitrogen, Dan11mag; 1:200), anti-CD69 (Invitrogen, H1.2F3; 1:20), anti-T-bet (Invitrogen, 4B10; 1:200), anti-IL-17A (Invitrogen, eBio17B7; 1:200), anti-P-S6 ribosomal protein (S235/236) (Cell Signaling, 8520S; 1:200), anti-CD25 (Invitrogen, PC61.5; 1:200), anti-granzyme B (Invitrogen, NGZB; 1:200), anti-CD8a (Invitrogen, 53-6.7; 1:200), anti-TNF-α (BD, MP6-XT22;1:200), TB10.4_4−11_ H2^Kb^ tetramer (Proimmune, F764-2A-G; 1:20), 32a_309–319_/D^b^ tetramer (MBL, TS-M549-2; 1:20), MitoTracker Green FM (Thermo Fisher, M7514; 1:1,000), fixation/permeabilization kit (BD Biosciences) and Cell Stimulation Cocktail (plus protein transport inhibitors) (500X) (Thermo Fisher, 00-4975-93). The following reagents were used for ELISA: mouse IL-2 Valukine ELISA kit (R&D SYSTEMS, VAL602) and LEGEND MAX mouse IFN-γ ELISA kit (Biolegend, 430807).

### Mouse strains

C57BL/6, Cd4^Cre^, *Ifng*^*fl/fl*^, *WDR24*^*fl/fl*^ and *Rag1*^−/−^ mice were purchased from Cyagen Biosciences. All genetic models were of the C57BL/6 background.

TB10Rg3 mice were generated using CRISPR knock-in technology in collaboration with Cyagen Biosciences. In brief, the guide RNA targeted mouse Hipp11 (H11) locus, the donor vector containing ‘H2-k^b^ Promoter-Kozak-TCR4-Human HBB polyA-IgH enhancer’ cassette, and Cas9 mRNA were co-injected into fertilized mouse eggs to generate targeted knock-in offspring. F_0_ founder animals were identified by PCR followed by sequence analysis, and bred to wild-type mice to test germline transmission and F_1_ animal generation (Supplementary Fig. 1). The T cell receptor of TB10Rg3 mice was designed to recognize the TB10.4_4–11_ antigen epitope of *Mtb* in the context of H2-k^b^. Knock-in Kozak-TCR4 sequences refer to a previous description^69^. All mice were bred in specific pathogen-free conditions at the Shanghai Pulmonary Hospital Laboratory Animal Center (temperature between 20 and 26 °C, relative humidity between 50% and 60%, light intensity in the feeding room 15–20 lx with 12 h:12 h light:dark cycle) and fed with 25 kGy irradiated feed (SLAC, P1101F) in accordance with hospital guidelines.

### Mice infection

**Extended Data Fig. 8:**
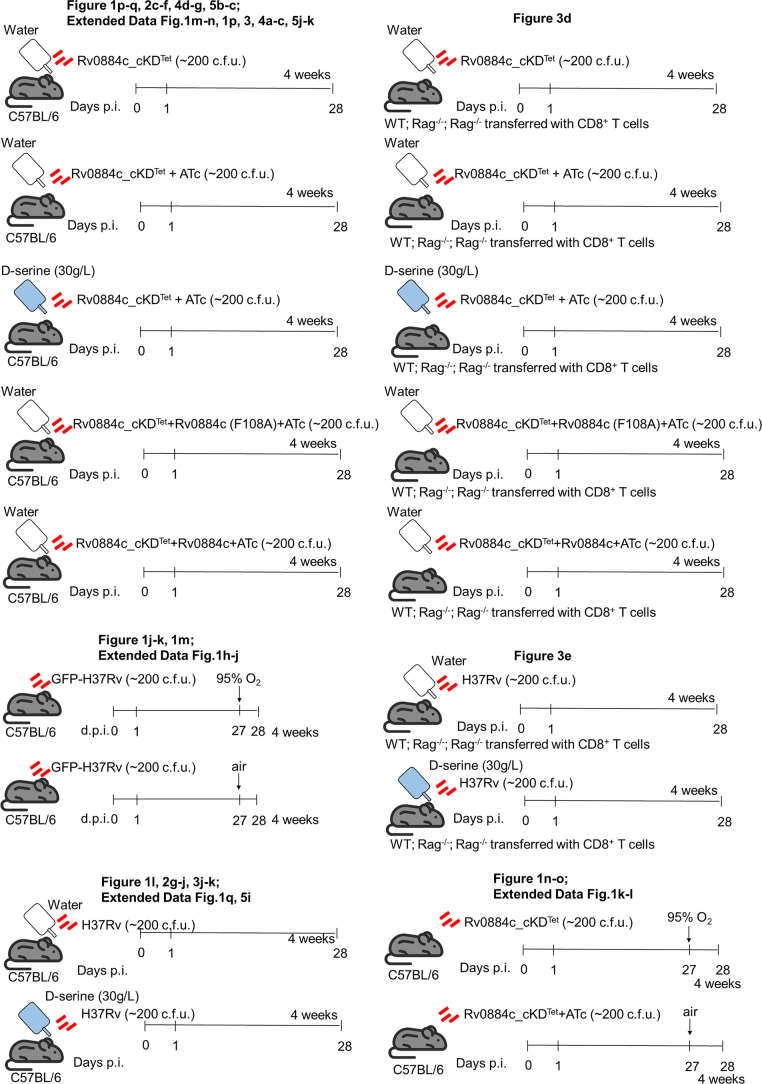
Schematic representation of mice experimental design. Schematic representation of mice experimental design for Figs. 1p–q, 2c–f, 4d–g and 5b,c, and Extended Data Figs. 1m,n,p, 3, 4a–c and 5j,k (left panels of top groups). Schematic representation of mice experimental design for Fig. 1j,k,m and Extended Data Fig. 1h–j (left panels of middle groups). Schematic representation of mice experimental design for Figs. 1l, 2g–j and 3j,k, and Extended Data Figs. 1q and 5i (left panels of bottom groups). Schematic representation of mice experimental design for Fig. 3d (right panels of top groups). Schematic representation of mice experimental design for Fig. 3e (right panels of middle groups). Schematic representation of mice experimental design for Fig. 1n,o, and Extended Data Fig. 1k,l (right panels of bottom groups).

C57BL/6 female mice, CD4^Cre^*Ifng*^*fl/fl*^ female mice, CD4^Cre^*WDR24*^*fl/fl*^ and *Rag1*^−/−^ female mice (6–8 weeks old) were divided randomly into cages and infected using an aerosol method with ~200 c.f.u.s of different H37Rv strains for 1 day or 4 weeks (using an inhalation exposure system from Glas-col) at the Biosafety Level-3 (BSL-3) Laboratory. Then the infected mice were treated with or without d-serine (30 g l^−1^) (Sigma, ≥98%) via drinking water, or fed a diet with or without doxycycline. Anti-CD45 antibody 30F11 (eBioscience, 16-0451-85) was given in 4 consecutive daily injections (1 μg g^−1^ body weight each) before the mice were killed to deplete peripheral blood leucocytes^70^. Mice were killed 1 day or 4 weeks after infection, as illustrated in Extended Data Fig. 8. All mice were age-, weight- and sex-matched in each experiment.

Serum of mice infected for 4 weeks was collected and used to detect the level of d-serine, l-serine or IL-2 according to manufacturer instructions as described below. Lung tissues of mice infected for 1 day or 4 weeks were collected and used to detect bacterial burden. Lung tissues were also used to analyse the percentage of various T cells, the level of d-serine or l-serine and complete the histological analysis. Draining lymph nodes of mice infected for 4 weeks were collected and used to analyse the percentage of various T cells and complete the histological analysis.

### C.f.u. assay

Parts of lung tissues were homogenized in 1 ml of PBS. Homogenates in 10-fold serial dilutions were plated on 7H10 agar supplemented with 10% OADC enrichment medium and incubated at 37 °C. Colonies were counted after 4 weeks of incubation at 37 °C in 5% CO_2_ and displayed on a log scale.

### Histopathology analysis

Part of lung tissues and dLNs were fixed in 4% neutral-buffered paraformaldehyde solution for 24 h and then embedded in paraffin. A series of sections with a thickness of 4–7 μm were then cut and stained with hematoxylin and eosin (H&E) or Ziehl–Neelsen (acid-fast bacillus) stain, in accordance with standard protocols. Imaging was performed using an automated whole-slide scanning device (3DHISTECH, Sysmex). Individual H&E slides were scored in a single-blinded fashion with a maximum score of 4 using leucocyte infiltration, haemorrhage, alveolar wall thickening and alveolar oedema as scoring indicators (Supplementary Fig. 2).

### Immunofluorescence staining with Hypoxyprobe-1 (PIMO)

C57BL/6 female mice (6–8 weeks old) were divided randomly into cages and infected using an aerosol method with ~200 c.f.u.s of GFP-H37Rv for 4 weeks (using an inhalation exposure system from Glas-col) at the BSL-3 Laboratory. Parts of infected mice were exposed to 95% O_2_ by the addition of carbogen for 20 h before killing. Hypoxyprobe-1 solution (Hypoxyprobe) was injected intraperitoneally at a dose of 60 mg kg^−1^ body weight 30 min before killing the mice. Immunofluorescence staining with Hypoxyprobe-1 (red) (Hypoxyprobe), F4/80 (Invitrogen, SP115) (pink) and DAPI (blue) was performed on frozen sections of the dLNs and lung tissues of GFP-H37Rv-infected C57BL/6 mice, following manufacturer instructions of the Hypoxyprobe-1 kit (Hypoxyprobe). Imaging was performed using an automated whole-slide scanning device (3DHISTECH, Sysmex) (Supplementary Fig. 2).

### Wayne’s in vitro hypoxia model

Conical screw-capped Nephelo flasks with 20 mm side arms and flat bases (Wheaton) were used to culture bacteria. *Mtb* H37Rv was grown to mid-log phase (OD_590_ ~0.4, estimated at 2.5 × 10^8^ c.f.u.s ml^−1^). For aerobic experiments, 200 ml of medium was inoculated with 2 ml of the culture and incubated at 37 °C on a magnetic stirrer set to rotate at 180 r.p.m. Simultaneously, 400 ml of medium was inoculated with 4 ml of identical culture in a tightly capped flask, placed on a tissue culture magnetic stirrer set at 70 r.p.m. and incubated at 37 °C to avoid perturbation of the surface. Samples for the quantitative proteomics assay of cell lysates and comparative metabolomics analysis of culture supernatant were taken after 14 days of vigorous aeration of cultures (Aeration) or 14 days of hypoxic cultures (Hypoxia)^22^.

For western blotting, qPCR detection or d-serine/l-serine quantitation assay of bacterial cultures, *Mycobacteria* were grown to mid-log phase as before. For aerobic conditions, 4 ml of protein-free liquid medium in 20 mm screw-capped culture tubes was inoculated with 0.4 ml of the culture and incubated at 37 °C in a shaker-incubator set to rotate at 150 r.p.m. Simultaneously, 8 ml of medium was inoculated with 0.8 ml of identical culture in tightly capped tubes, placed on a shaker-incubator set to rotate at 70 r.p.m. and incubated at 37 °C to avoid perturbation of the surface. Then 4 ml cultures under aeration or hypoxia after indicated days were individually collected and centrifuged to separate the culture supernatant and precipitated bacteria cells. For samples at day 0, identical *Mycobacteria* cultures were added in the aeration or hypoxia culture tubes similarly, and the culture supernatant and precipitated bacteria cells were collected immediately.

### Comparative metabolomics analysis using GC–MS

The culture supernatant and collected cells were separated via filtration through a membrane with 0.22 μm pore (Millipore). Culture supernatant samples of *Mtb* were transferred to Eppendorf tubes with methanol (for the first global metabolomics analysis, Fig. 1a) or acetonitrile:isopropanol:water (3:3:2, v/v/v) (for the second global metabolomics analysis, Extended Data Fig. 1o), and 10 μl of internal standard (adonitol, 0.5 mg ml^−1^ stock) was added and mixed by vortexing for 30 s followed by ultrasonication for 10 min in an ice bath. The samples were centrifuged at 4 °C and 12,000 *g* for 15 min. The supernatant was transferred into an Eppendorf tube and 60 μl of each sample was removed and mixed with quality control (QC) samples. The samples were dry extracted in a vacuum concentrator. After evaporation, 50 μl of methoxyamination hydrochloride (20 mg ml^−1^ in pyridine) was added and the samples were incubated at 80 °C for 30 min and then derivatized with BSTFA reagent (1% TMCS, v/v) at 70 °C for 1.5 h. The samples were gradually cooled to room temperature and 5 μl of fatty acid methyl esters (in chloroform) was added to the QC sample. All samples were then analysed using GC coupled with a time-of-flight MS. Raw data analysis, including peak extraction, baseline adjustment, deconvolution, alignment and integration, was performed using Chroma TOF (v.4.3x, LECO) software. The LECO-Fiehn Rtx5 database^54^ and the updated Fiehn library database^57^ were used for metabolite identification by matching mass spectra and retention indices. Finally, peaks detected in less than half of QC samples or with relative standard deviation (RSD) > 30% in QC samples were removed.

### Quantitative comparative proteomics profiling

The cell lysate samples for proteomics analysis were sonicated three times on ice using a high-intensity ultrasonic processor (Ningbo Scientz Biotechnology) in lysis buffer (8 M urea, 1% protease-inhibitor cocktail). The remaining debris was removed by centrifugation (12,000 *g*, 4°C, 10 min). Finally, the supernatant was collected and the protein concentration was determined using a 2-D Quant kit according to manufacturer instructions. Proteins were digested with trypsin. The peptides were extracted with 0.5% formic acid and 50% acetonitrile, followed by 0.1% formic acid and 80% acetonitrile. The extracted peptides were bound to a Magic C18 AQ reverse phase column (100 μm × 50 mm; Michrom Bioresources). An Agilent 1100 binary pump was used to generate the HPLC gradient as follows: 0–5% B for 5 min, 5–45% B for 40 min, 45–80% B for 3 min and then to 5% B in 2 min (A = 0.1 M acetic acid in water; B = 0.1 M acetic acid and 70% acetonitrile). The eluted peptides were sprayed into an LTQ Orbitrap XL MS (Thermo Electron) equipped with a nano-ESI ion source. The MS was operated in data-dependent mode, automatically switching between MS and tandem MS acquisition. The resulting MS/MS data were processed using the Mascot search engine (v.2.3.0). Tandem MS were searched against the *Mtb* H37Rv database (6,168 sequences) or the Uni-ProtKB/Swiss-Prot database. Database searches were performed using Xcalibur software with Mascot. The resulting Proteome Discoverer Report contained all assembled proteins with peptide sequences, possible post-translational modifications (for example, phosphorylation) and matched spectrum counts.

### d-serine and l-serine quantitation assay

The d-serine concentration of culture supernatants from different *Mtb* strains incubated under aeration or hypoxia, culture supernatants and cell lysates derived from iBMDMs infected with different *Mtb* strains, as well as lung homogenate supernatants and sera derived from mice infected with different *Mtb* strains or under different conditions were detected according to the mouse d-serine ELISA kit manufacturer instructions (MyBioSource).

The l-serine concentration of culture supernatants from different *Mtb* strains incubated under aeration or hypoxia, lung homogenate supernatants and sera derived from mice infected with different *Mtb* strains or under different conditions were detected according to the mouse l-serine ELISA kit manufacturer instructions (ImmuSmol).

### Construction of *Mycobacteria* strains

The Rv0884c CRISPRi strains were constructed in *Mtb* H37Rv. We designed the specific small guide RNA (sgRNA) (gtcgcggggtttgatggcgg) targeting the non-template strand of Rv0884c and then cloned the annealed products into the pLJR965 plasmid, which harbours a tetracycline-inducible dcas9, named pLJR965-sgserC. The Rv0884c-complemented strain was constructed by cloning the Rv0884c allele containing synonymous mutations at the sgRNA target location into the pLJR965-sgRv0884c plasmid, named pLJR965-sgRv0884c-Rv0884c. The Rv0884c (F108A)-mutant strain was constructed by mutating the phenylalanine (F) 108 residue of the Rv0884c allele to alanine (A) and cloning into the pLJR965-sgRv0884c-Rv0884c plasmid, named pLJR965-sgRv0884c-Rv0884c (F108A). These plasmids were then transformed into *Mtb* H37Rv. After electroporation, the transductants were plated on selective medium, 7H10 agar containing 10% ADC enrichment medium and 50 μg ml^−1^ kanamycin, and cultured at 37 °C. Kanamycin-resistant clones were isolated, analysed by PCR for the presence of the dcas9 cassette and tested for the efficiency of knockdown by monitoring the growth of tested strains on 7H10 agar in the presence of the ATc inducer. Western blotting was used to confirm the knockdown of Rv0884c with anti-Rv0884c polyclonal antibody at a 1:1,000 dilution; anti-SigA monoclonal antibody at a 1:1,000 dilution was used as the reference antibody^71^.

### Growth curve

*Mycobacteria* were grown to mid-log phase in 7H9 broth with 10% OADC, 0.05% Tween-80 and the required antibiotics. Growth curves for each strain were determined using a Bioscreen C Growth Curve Instrument (Labsystems Oy) and a honeycomb plate with 100 wells (Labsystems Oy). Briefly, 200 μl of each bacterial suspension, adjusted to a similar density, was added to each well and cultured with shaking at 37 °C for H37Rv. The OD_590_ was measured every day. Hypoxic conditions were established by covering each culture with 50 μl of paraffin oil. Cultures were incubated at 37 °C for 14 days for H37Rv. Three independent experiments were performed, each in triplicate.

### Intracellular bacterial growth in BMDMs

BMDMs were infected with *Mtb* at a multiplicity of infection (MOI) of 5. At 3 h, 24 h or 96 h post infection, BMDMs were lysed with 0.2% Triton X-100, and lysates in 10-fold serial dilutions were plated on 7H10 agar supplemented with 10% OADC enrichment medium and incubated at 37 °C. Colonies were counted after 4 weeks of incubation at 37 °C in 5% CO_2_ (ref. ^72^) and displayed on a linear scale.

### qPCR analysis

qPCR analysis was performed using gene-specific primers (*Rv0884c*-F: GGCTATGAGGTGATACTGGG, *Rv0884c*-R: GGCGTCGATGACGACCAAGG; *sigA*-F: GATGACCGAGCTTAGCGAGC, *sigA*-R: CGTAGGTGGAGAACTTGTACC). Total RNA was extracted with 1 ml of Trizol reagent according to manufacturer instructions (Thermo Fisher). Total RNA (1 µg) was used for complementary DNA synthesis with the ReverTra Ace qPCR RT kit (Toyobo). qPCR was performed using the SYBR RT–PCR kit (Toyobo) in an LC480 thermocycler (Roche). The 2^−ΔΔCt^ method was adopted to analyse relative gene expression, which was normalized to the expression of *sigA*. qPCR data were collected from at least three independent experiments with three technical replicates per experiment.

### Luciferase assay

The dual-luciferase reporter assay system (Promega) was used to detect T-bet transcription. The T-bet promoter sequence (2 kb upstream of the T-bet coding DNA sequence) was cloned into the PLG3-Basic vector. *Renilla* luciferase controlled by pRL-TK vector was constitutively expressed and served as the internal control. HEK293T cells were transiently transfected with PLG3-Basis-T-bet promoter and pRL-TK vector, and then treated with PBS or d-serine (10 mM, Sigma) (purity ≥98%) for 48 h. Experiments were performed according to standard protocols^63^.

### Confocal microscopy

The cell samples were fixed with 4% paraformaldehyde for 30 min, permeabilized with 0.1% Triton X-100 for 3 min and blocked with 3% BSA in PBS for 15 min at room temperature. Cells were subsequently stained with rabbit anti-mTOR (7C10, Cell Signaling Technologies) and mouse anti-LAMP2 (MA1-205, Thermo Fisher) overnight at 4 °C, followed by detection with goat anti-rabbit IgG (H + L) highly cross-adsorbed secondary antibody, Alexa Fluor 488 (Thermo Fisher, A-11034) and goat anti-mouse IgG (H + L) highly cross-adsorbed secondary antibody, Alexa Fluor 568 (Thermo Fisher, A-11031). Nuclei were stained with DAPI. Samples were visualized using a Leica confocal microscope.

### Preparation of anti-Rv0884c polyclonal antibody

The rabbit polyclonal antibody to Rv0884c was generated by immunization of rabbits with the purified Rv0884c fusion protein, in collaboration with ABclonal Biotech.

### Immunoprecipitation and immunoblotting

HEK293T cells were transiently transfected with different plasmids using Lipofectamine 2000. After 48 h, cells were lysed in cell-lysis buffer for western blotting and immunoprecipitation (Beyotime) with 1% protease-inhibitor cocktail (P8340, Sigma). After incubating for 30 min on ice, the lysates were centrifuged (12,000 *g*, 10 min, 4 °C) to remove cellular debris. A 50 μl aliquot of the clarified lysate was prepared as the total cell lysate sample. The remaining lysates were incubated with anti-FLAG or anti-HA magnetic beads (MCE) overnight at 4 °C for immunoprecipitation. To confirm the interaction of d-serine with WDR24, the remaining cell lysate was incubated with d-serine-biotin, l-serine-biotin or biotin (100 μM) (Bootech Bioscience) for 2 h at 4 °C and then incubated with streptavidin magnetic beads (MCE, HY-K0208) overnight at 4 °C for immunoprecipitation. For endogenous immunoprecipitation, the cells were lysed, the lysates were incubated with anti-WDR24 antibody (rabbit IgG used as control) and protein A/G magnetic beads (MCE) overnight at 4 °C, washed five times with 0.1% Tween-20 and boiled with SDS loading buffer for 10 min^68^.

Standard western blotting procedures were used as before. In brief, cell extracts of *Mtb*, T cells or HEK293T cells, Rv0884c_107–376aa_ or GST-WDR24, and GST proteins were denatured in 1× SDS protein sample buffer and separated on 10% or 12% SDS-polyacrylamide gels. The gels were then transferred to nitrocellulose membranes. Next, the membranes were blocked, incubated with primary antibodies and washed three times before incubation with the secondary antibody. The concentration of the primary antibodies was 1:1,000 for anti-SigA, anti-Rv0884c, anti-p-S6K1, anti-S6K1, anti-Sec13 and anti-WDR24. The concentration of the primary antibodies was 1:5,000 for anti-GAPDH and anti-β-actin. Horseradish peroxidase-conjugated goat anti-rabbit or anti-mouse polyclonal antibody was used as the secondary antibody at a 1:5,000 dilution. After a final wash, analysis was conducted using ECL reagent (Thermo Fisher).

### T cell purification, activation, differentiation and stimulation

#### Purification, activation, differentiation and stimulation of CD8^**+**^ T cells or CD4^**+**^ T cells derived from C57BL/6 mice

Naïve CD8^+^ T cells or naïve CD4^+^ T cells were isolated from spleens of C57BL/6 mice using a naive CD8^+^ T cell or naive CD4^+^ T cell isolation kit according to manufacturer instructions (BioLegend). IFN-γ^−/−^ CD8^+^ T cells were similarly derived from spleens of CD4^Cre^*Ifng*^*fl/fl*^ mice. Purified naïve CD8^+^ T cells or naïve CD4^+^ T cells were activated in vitro for 24 h with 5 µg ml^−1^ plate-bound anti-CD3 (Peprotech; concentration 5 mg ml^−1^) in the presence of 5 μg ml^−1^ anti-CD28 antibodies (Peprotech; concentration 5 mg ml^−1^) and 20 ng ml^−1^ human IL-2 (Peprotech; concentration 10 mg ml^−1^). Activated CD8^+^ T cells were used to construct WDR24-knockdown cells (T cell viral transduction) and complete the co-culture assay. Then, activated CD4^+^ T cells or CD8^+^ T cells were differentiated towards IFN-γ^+^ T cells in vitro for 4 days via culture in complete RPMI 1640 medium supplemented 10% (v/v) FBS and 1% (v/v) penicillin–streptomycin^32,73^ in the presence of 20 ng ml^−1^ human IL-2 (Peprotech; concentration 10 mg ml^−1^) and 10 ng ml^−1^ mouse IL-12 p70 (Peprotech). Meanwhile, cells were treated with Mock, d-serine (10 mM) or l-serine (10 mM, Sigma) (purity ≥99%) to detect whether d-serine inhibited the differentiation of CD4^+^ or CD8^+^ T cells. Rapamycin (25 nM), 2-DG (4 mM) and oligomycin (1 μM) were used to treat the CD8^+^ T cells and detect whether d-serine inhibited the CD8^+^ T cells through the corresponding signalling pathway.

#### Purification, activation, differentiation and stimulation of CD8^**+**^ T cells derived from TB10Rg3 mice

After 24 h of intravenous injection of peptide TB10.4_4–11_ into TB10Rg3 mice, activated CD8^+^ T cells were isolated from spleens of TB10Rg3 mice using a CD8^+^ T cell isolation kit according to manufacturer instructions (BioLegend), and used for co-culture assay.

### T cell viral transduction

After CD8^+^ T cell activation, viral transduction was performed by spin-infection at 1,000 *g* with 8 ng ml^−1^ polybrene (Sigma-Aldrich) for 2 h at 37 °C, followed by a 3 h rest at 37 °C and 5% CO_2_. Cells were washed and incubated with mouse IL-2 (10 ng ml^−1^; Peprotech) for 2 days. Successfully transfected CD8^+^IFN-γ^+^ T cells were obtained and used for functional analysis. The sequence of shRNA targeting WDR24 was 5′CTTCATGAAGTGCTTTGACCT3′; 3′CATCTTCTTTAAGCGCAAGCT5′.

### T cell and macrophage co-culture assay

CD8^+^ T cells were derived from TB10Rg3 mice or C57BL/6 mice, and IFN-γ^−/−^ CD8^+^ T cells were derived from CD4^Cre^*Ifng*^fl/fl^. Activation, genetic manipulation (T cell viral transduction) and culture were conducted as described above.

BMDMs at 1 × 10^5^ per well were infected with different *Mtb* strains (MOI = 5). After 3 h, extracellular bacteria were removed by washing three times with warm PBS. Infected BMDMs were incubated at 37 °C for an additional 21 h and parts of the BMDMs were pulsed by incubating with 10 μM TB10.4 peptides (IMYNYPAM) for 1 h. Unbound peptides were washed off. Next, activated CD8^+^ T cells derived from C57BL/6, CD4^Cre^*Ifng*^*fl/fl*^ or TB10Rg3 mice and treated with or without d-serine (10 mM) were continuously co-cultured with BMDMs at 5 × 10^5^ per well for indicated times. Percentage of CD69^+^CD8^+^ T cells, level of IFN-γ and intracellular bacterial growth were then detected^36,39,74^.

#### Percentage of CD69^**+**^CD8^**+**^ T cells assay

After 2 h of co-culture, percentage of CD69^+^ TB10Rg3 T cells was measured in TB10Rg3 T cells using FACS.

#### IFN-γ level assay

After co-culture for 3 days, the level of IFN-γ in TB10Rg3 T cells was measured using ELISA.

#### Intracellular bacterial growth assay

After 1 day of infection with *Mtb* strains, pulsed or unpulsed BMDMs were co-incubated with CD8^+^ T cells for 3 days and intracellular c.f.u. in BMDMs infected for 1 or 4 days were determined. BMDMs infected with *Mtb* strains and pulsed with TB10.4_4−11_ peptide were indicated as ‘+’. BMDMs infected with *Mtb* strains for 1 day were indicated as ‘d1’. Unpulsed BMDMs infected with *Mtb* strains for 1 day and further incubated without CD8^+^ T cells for another 3 days were indicated as ‘No T’. Unpulsed BMDMs infected with *Mtb* strains for 1 day and then co-incubated with CD8^+^ T cells for another 3 days were indicated as ‘Rg3’.

### Seahorse metabolic assay

OCR and ECAR were measured using the Seahorse XF Cell Mito Stress Test kit (Agilent) following manufacturer instructions. In brief, naïve CD8^+^ T cells obtained from mice spleens were stimulated with plate-bound anti-CD3 in the presence of anti-CD28 antibodies and IL-2 for 24 h and then treated with PBS, d-serine or l-serine (10 mM). Activated CD8^+^ T cells (2.5 × 10^5^) were suspended in XF medium and subsequently seeded in a poly-l-lysine-coated XF96 plate. The OCR and ECAR under basal conditions and in response to 1 μM oligomycin, 1.5 μM carbonyl cyanide 4-(trifluoromethoxy) phenylhydrazone and 500 nM rotenone were measured using an XF96 Extracellular Flux Analyzer (Seahorse Bioscience).

### Flow cytometry

To analyse surface markers, cells were stained in PBS (Gibco) containing 2% (w/v) BSA (Sigma). Surface proteins were stained for 30 min on ice. For phospho-flow cytometry analysis, to preserve the fluorescent proteins, cells were fixed with 2% paraformaldehyde for 30 min at room temperature, followed by permeabilization with 90% ice-cold methanol for 30 min and then staining in 1× permeabilization buffer (eBioscience) for 30 min at room temperature. Transcription factor staining was performed with FOXP3/transcription factor staining buffers according to manufacturer instructions (eBioscience). Intracellular staining for cytokines was performed with a fixation/permeabilization kit (BD Biosciences)^75^. In brief, cells were activated using the Cell Stimulation Cocktail (eBioscience, 00-4975-93) and then stained with surface markers for 30 min, fixed, permeabilized using fixation/permeabilization kit (BD Biosciences) and stained with anti-IFN-γ (Biolegend, XMG1.2), anti-IL-17A (Invitrogen, eBio17B7), anti-granzyme B (Invitrogen, NGZB) and anti-TNF-α (BD, MP6-XT22) antibodies. Cells were washed twice and the frequencies of CD8^+^IFN-γ^+^ T cells, IL-17A^+^ CD8^+^ T cells, granzyme B^+^ CD8^+^ T cells and TNF-α^+^ CD8^+^ T cells were determined by flow cytometry. The determination of mitochondrial mass was conducted using flow cytometry according to manufacturer instructions (Thermo Fisher, M7514). Fluorescence minus one (FMO) controls were used for accurately discriminating positive versus negative signals of CD69 and Eomes staining. The full gating strategy is illustrated in Supplementary Fig. 3.

### Pull-down and mass spectrometry analysis

Jurkat cells were lysed using RIPA lysis buffer (Beyotime) and supplemented with protease cocktail (P8340, Sigma-Aldrich) and 1 mM of phenyl methane sulfonyl fluoride. Parts of the lysates were centrifuged at 12,000 *g* for 10 min and the cellular debris was discarded. The remaining whole-cell lysates were mixed with biotin, biotin-l-serine or biotin-d-serine (100 μM) at 4°C for 4 h and then incubated with streptavidin magnetic beads (MCE, HY-K0208) at 4 °C overnight. The beads were then washed and boiled with 1× SDS loading buffer for SDS–PAGE analysis, followed by Coomassie blue staining for liquid chromatography (LC)–MS analysis, or the proteins were transferred onto nitrocellulose membrane and incubated with antibodies. The protein gel was destained, reduced, alkylated, digested with trypsin and analysed using MS. The resulting MS data were processed with the Mascot search engine (v.2.3.0). Tandem MS were searched against the Uni-ProtKB/Swiss-Prot database.

### Pull down in vitro

GST-WDR24 (2 μg, Proteintech, Ag14774) or GST proteins (Sigma, 50812-37-8) were added to cytosolic buffer (40 mM HEPES pH 7.4, 0.1% Triton X-100, 10 mM NaCl, 150 mM KCl, 2.5 mM MgCl_2_, 1 mM GDP) containing 10 mM of biotin, biotin-d-serine or biotin-l-serine and incubated with streptavidin magnetic beads for 6 h at 4 °C. The beads were centrifuged, washed with cytosolic buffer and denatured by heating at 100 °C in loading buffer. Proteins were separated by SDS–PAGE gel and assayed by Coomassie staining and immunoblotting^8^.

### Statistical analysis

All experiment data were analysed using GraphPad Prism 9.0 software. Data distribution was assumed to be normal, but this was not formally tested. Data collection was randomized and animals/samples were assigned to the various experimental groups randomly. The data collection and analysis were not performed blind to the conditions of the experiments, except for the H&E slides scoring. No animals or data points were excluded from the analyses for any reason. The statistical significance of differences between two groups was determined by two-tailed unpaired Student’s *t*-test. Two-way analysis of variance (ANOVA) with Tukey’s multiple comparisons test and one-way ANOVA with Tukey’s multiple comparisons test were used for statistical analysis when comparing more than 2 groups. *P* < 0.05 was considered significant. All data were expressed as mean ± s.e.m. of the averages of technical replicates from the indicated number of independent experiments. No statistical methods were used to pre-determine sample sizes, but our sample sizes are similar to those reported in previous publications^68^.

### Reporting summary

Further information on research design is available in the Nature Portfolio Reporting Summary linked to this article.

### Supplementary information

Supplementary Information Supplementary Figs. 1–3.

Reporting Summary

Supplementary Tables 1–4 Supplementary Table 1. The raw data of Fig. 1a. Table 2. The raw data of Fig. 1c. Table 3. The raw data of Extended Data Fig. 1o. Table 4. Bacterial strains and plasmids used in this study.

### Source data

Source Data Fig. 1 Statistical source data.

Source Data Fig. 2 Statistical source data.

Source Data Fig. 3 Statistical source data.

Source Data Fig. 4 Statistical source data.

Source Data Fig. 5 Statistical source data.

Source Data Fig. 6 Statistical source data.

Source Data Extended Data Fig. 1 Statistical source data.

Source Data Extended Data Fig. 2 Statistical source data.

Source Data Extended Data Fig. 3 Statistical source data.

Source Data Extended Data Fig. 4 Statistical source data.

Source Data Extended Data Fig. 5 Statistical source data.

Source Data Extended Data Fig. 6 Source data for IP–MS analysis of the proteins co-immunoprecipitating with biotin, biotin-d-serine and biotin-l-serine.

Source Data Figs. 1, 5 and 6, and Extended Data Figs. 1, 5 and Fig. 6 Unprocessed western blots and/or gels.

## Data Availability

Proteomics data sets for quantitative comparative proteomics profiling can be accessed in the PRIDE Database under project accession number: PXD050258. In addition, the metabolomics data for comparative metabolomics analysis and proteomics data analysis are provided as Supplementary Tables 1–3. The other primary source data are provided with this paper. Original western blot images are provided as Source data and full scans for images as Supplementary Fig. 2. Source data are provided with this paper.

## Code availability

All analyses were done reproducibly using publicly available R scripts.
