# Supplementary material for: Mycobacterium tuberculosis produces d-serine under hypoxia to limit CD8+ T cell-dependent immunity in mice
Source: Nat Microbiol. 2024 May 28;9(7):1856–72. doi: 10.1038/s41564-024-01701-1 (PMC11222154; doi:10.1038/s41564-024-01701-1)
Supplement: Supplementary file 1 — Supplementary Figs. 1–3. [file 41564_2024_1701_MOESM1_ESM.pdf]

nature microbiology

Supplementary information https://doi.org/10.1038/s41564-024-01701-1

Mycobacterium tuberculosis produces D-serine under hypoxia to limit CD8^{+} T cell-dependent immunity in mice

In the format provided by the authors and unedited

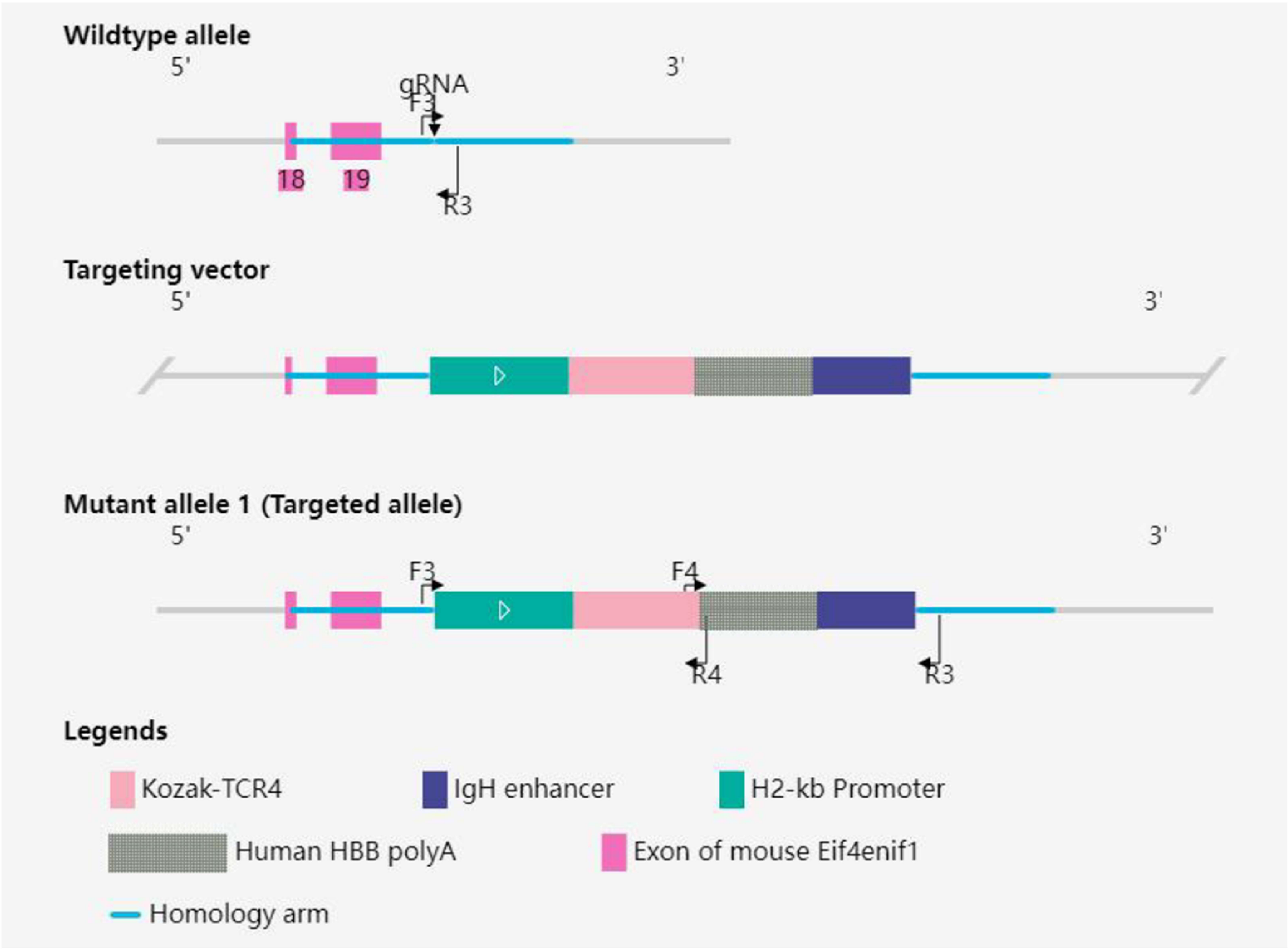

5'arm | KI

AAAAGCAGTCCCACTGGTCAGGAAAGGAACACTAGTGCACGGCGCGCCAAGCTTGGCCCACAAGGAGTGGCAATGTTAGG

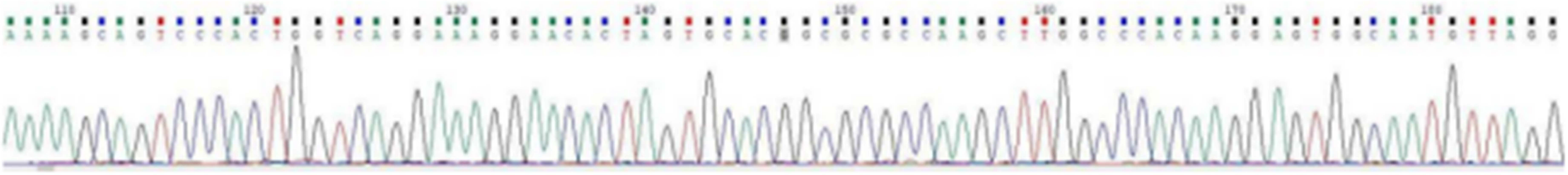

KI | 3'arm

GTAATGACCCAGACAGAGAAAGCCAGACTCATAACTCGAGTTATCCTGGGTGTCTGCTGAGCCAACAGTGGTAGTAAGGT

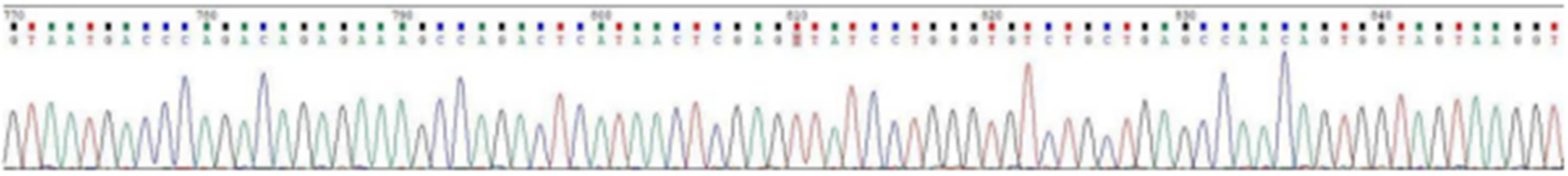

Supplementary Figure 1. Genotyping Strategy for TB10Rg3 mice

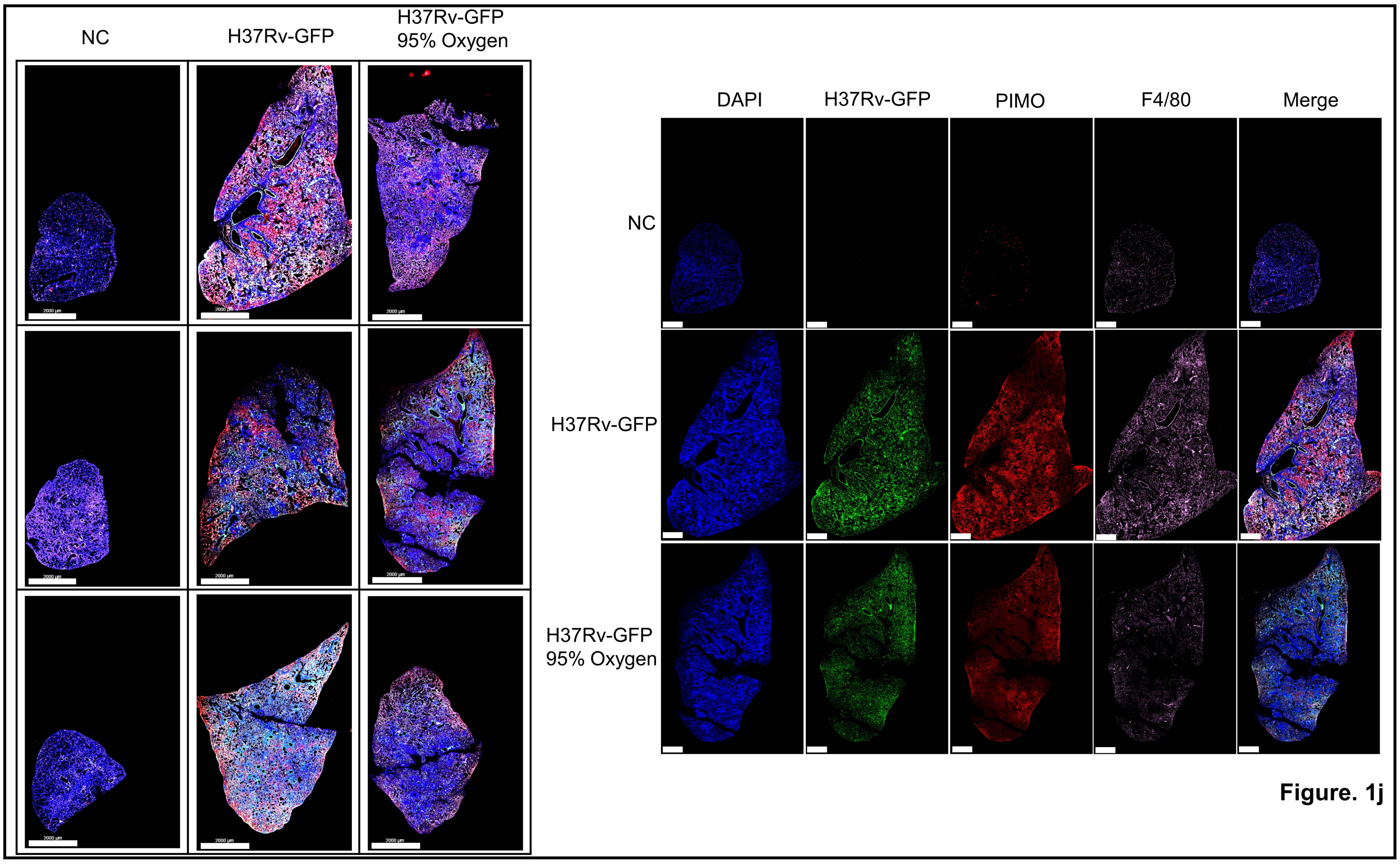

Figure. 1j

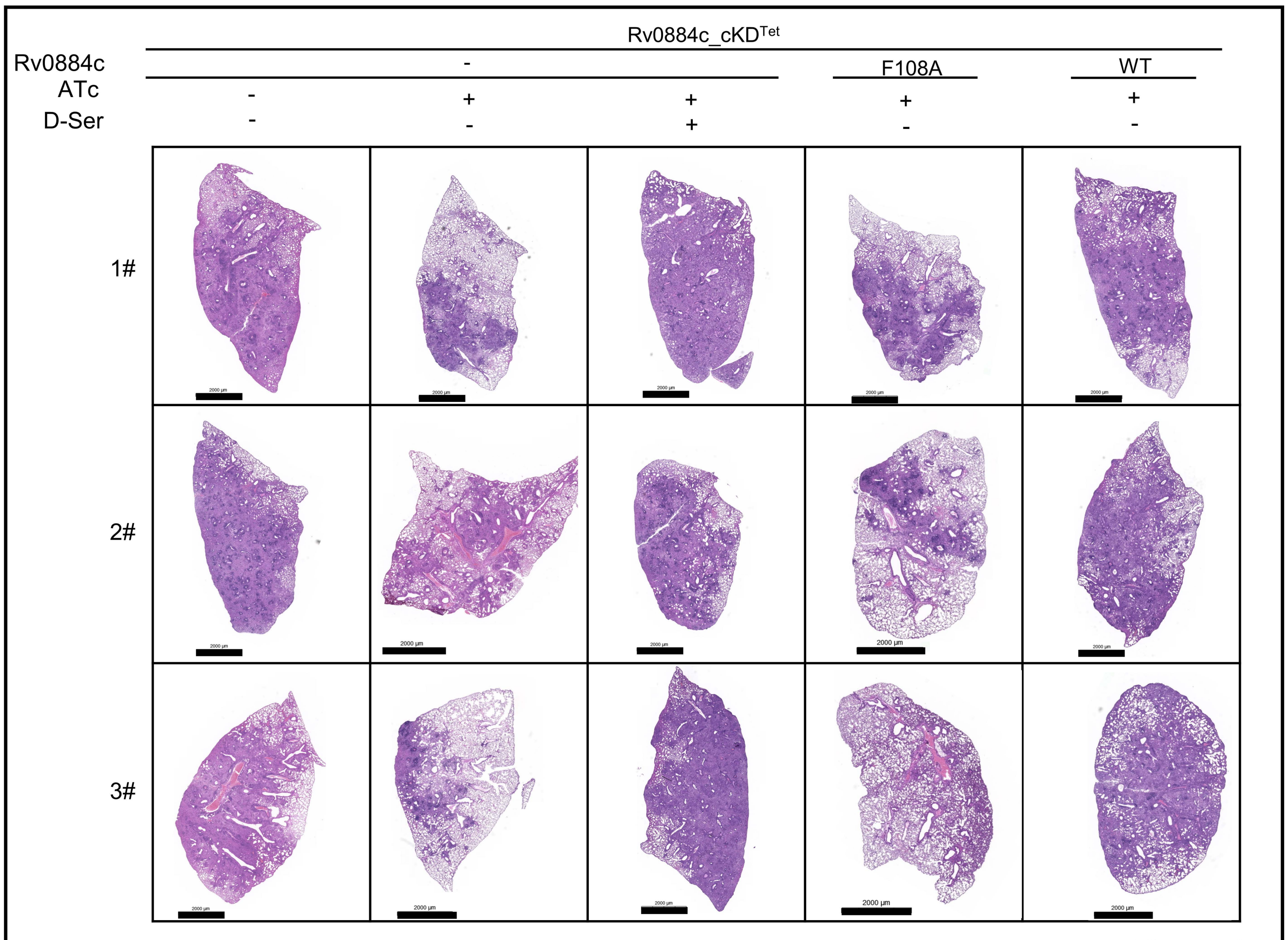

Figure. 2c

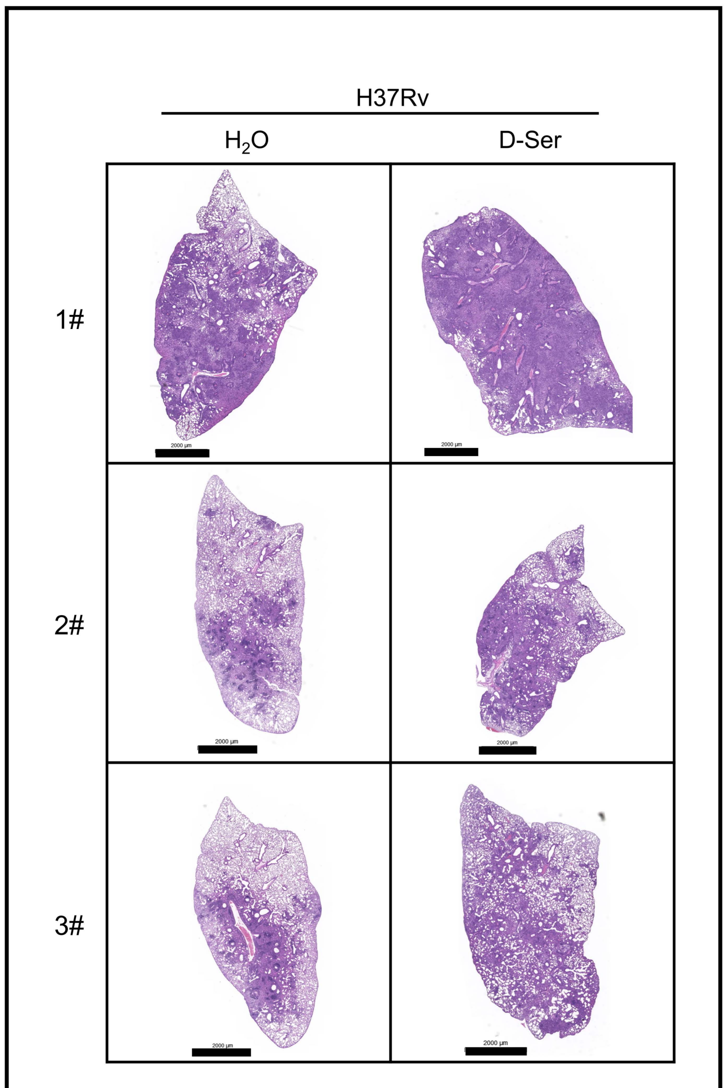

Figure. 2g

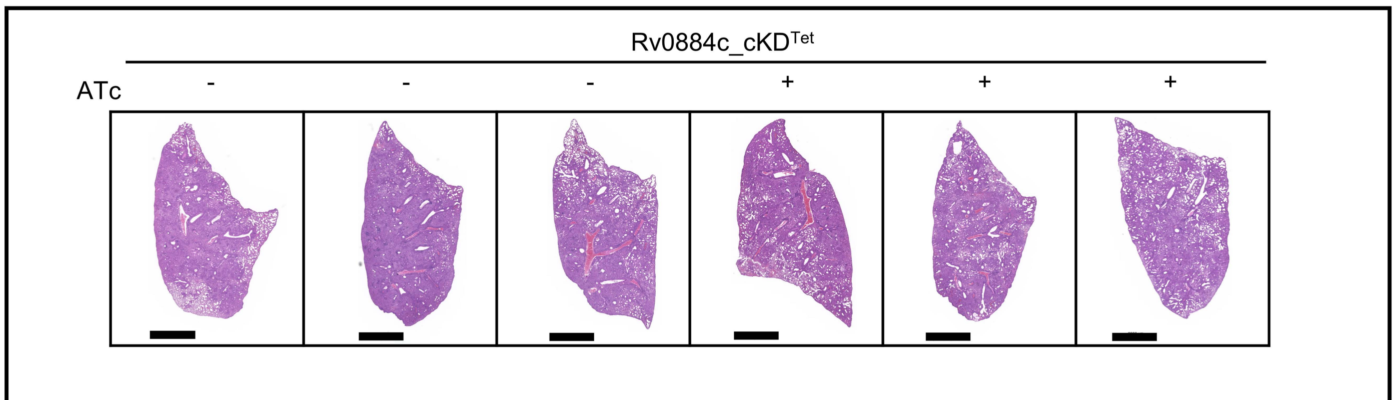

Figure. 4i

Supplementary Figure 2. Full-size micrographs of 3 lung tissues from each group of mice

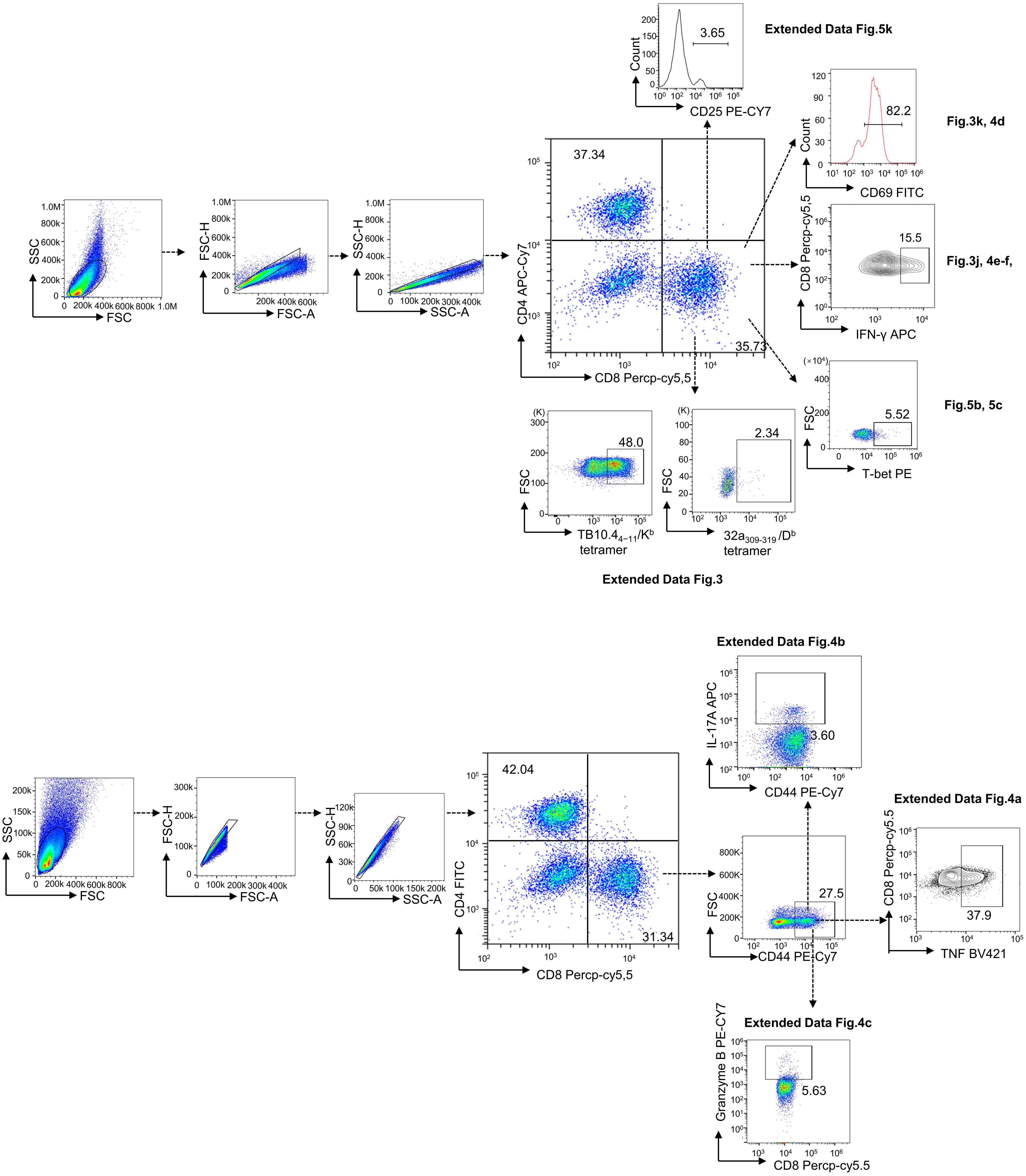

Supplementary Figure 3. Gate strategy for flow cytometry analysis in animal experiments
