# Supplementary material for: Mycobacterium tuberculosis produces d-serine under hypoxia to limit CD8+ T cell-dependent immunity in mice
Source: Nat Microbiol. 2024 May 28;9(7):1856–72. doi: 10.1038/s41564-024-01701-1 (PMC11222154; doi:10.1038/s41564-024-01701-1)
Supplement: Supplementary file 2 — Reporting Summary [file 41564_2024_1701_MOESM2_ESM.pdf]

nature portfolio

Corresponding author(s): Baoxue Ge, Hua Yang, Lilin Ye

Last updated by author(s): Mar 7, 2024

Reporting Summary

Nature Portfolio wishes to improve the reproducibility of the work that we publish. This form provides structure for consistency and transparency in reporting. For further information on Nature Portfolio policies, see our Editorial Policies and the Editorial Policy Checklist.

Statistics

For all statistical analyses, confirm that the following items are present in the figure legend, table legend, main text, or Methods section.

n/a	Confirmed	
☐	☒	The exact sample size (*n*) for each experimental group/condition, given as a discrete number and unit of measurement
☐	☒	A statement on whether measurements were taken from distinct samples or whether the same sample was measured repeatedly
☐	☒	The statistical test(s) used AND whether they are one- or two-sided<br>*Only common tests should be described solely by name; describe more complex techniques in the Methods section.*
☒	☐	A description of all covariates tested
☒	☐	A description of any assumptions or corrections, such as tests of normality and adjustment for multiple comparisons
☐	☒	A full description of the statistical parameters including central tendency (e.g. means) or other basic estimates (e.g. regression coefficient) AND variation (e.g. standard deviation) or associated estimates of uncertainty (e.g. confidence intervals)
☐	☒	For null hypothesis testing, the test statistic (e.g. *F*, *t*, *r*) with confidence intervals, effect sizes, degrees of freedom and *P* value noted<br>*Give P values as exact values whenever suitable.*
☒	☐	For Bayesian analysis, information on the choice of priors and Markov chain Monte Carlo settings
☒	☐	For hierarchical and complex designs, identification of the appropriate level for tests and full reporting of outcomes
☒	☐	Estimates of effect sizes (e.g. Cohen's *d*, Pearson's *r*), indicating how they were calculated

Our web collection on statistics for biologists contains articles on many of the points above.

Software and code

Policy information about availability of computer code

Data collection	The western blot data collection was performed with ImageQuant LAS 4000mini and Amersham Imager 600. Confocol data was obtained with Leica SP8 microscope. The flow cytometry data was obtained using BD FACSDiva and cytoflex cytexpert. The Seahorse data collection was performed using Agilent Seahorse_Wave_Desktop_v2_6_3_5. And qPCR data was obtained using LC480 thermocycler (Roche, Indianapolis, IN, USA). The metabolomics data collection was performed using GC-TOF-MS. The proteomics data collection was performed using LTQ Orbitrap XL MS (Thermo Electron Corp., Waltham, MA, USA) equipped with a nano-ESI ion source.
Data analysis	Data analysis of western blot was performed with Image J (V1.8.0). Statistical analysis of data was obtained using Prism Graphpad 9.0. Data analysis of flow cytometry was performed with FlowJo 10. Data analysis of metabolomics was performed with Chroma TOF (v. 4.3x, LECO Corp., Saint Joseph, MI, USA). Data analysis of proteomics was performed using XCalibur software with MASCOT.

For manuscripts utilizing custom algorithms or software that are central to the research but not yet described in published literature, software must be made available to editors and reviewers. We strongly encourage code deposition in a community repository (e.g. GitHub). See the Nature Portfolio guidelines for submitting code & software for further information.

Data

Policy information about availability of data

All manuscripts must include a data availability statement. This statement should provide the following information, where applicable:

- Accession codes, unique identifiers, or web links for publicly available datasets
- A description of any restrictions on data availability
- For clinical datasets or third party data, please ensure that the statement adheres to our policy

Proteomics data sets for quantitative comparative proteomics profiling can be accessed in the PRIDE Database under the project accession number: PXD050258. Additionally, the metabolomics data for comparative metabolomics analysis and proteomics data analysis have been deposited as Supplementary Tables S1, S2, and S3. The other primary source data have been provided with the manuscript. Original western blot images have been uploaded as Source Data and full scans for images have been uploaded as Supplementary Figures 2.

Human research participants

Policy information about studies involving human research participants and Sex and Gender in Research.

Reporting on sex and gender	not applicable
Population characteristics	not applicable
Recruitment	not applicable
Ethics oversight	not applicable

Note that full information on the approval of the study protocol must also be provided in the manuscript.

Field-specific reporting

Please select the one below that is the best fit for your research. If you are not sure, read the appropriate sections before making your selection.

☒ Life sciences ☐ Behavioural & social sciences ☐ Ecological, evolutionary & environmental sciences

For a reference copy of the document with all sections, see nature.com/documents/nr-reporting-summary-flat.pdf

Life sciences study design

All studies must disclose on these points even when the disclosure is negative.

Sample size	No statistical methods were used to predetermine sample sizes. Sample size was based on empirical data from pilot experiments. The corresponding number of events that was analyzed is indicated in the figure legends.
Data exclusions	No data were excluded from analysis.
Replication	We have indicated the number of independent experiments performed in the figure legends or the method.
Randomization	For mice infection experiments, mice were divided randomly into cages and infected with different H37Rv strains. H37Rv strains were allocated into groups according to genotype of interest.
Blinding	The investigators were blinded during data collection and analysis where possible. This included qPCR data collection and H&E slides score.

Reporting for specific materials, systems and methods

We require information from authors about some types of materials, experimental systems and methods used in many studies. Here, indicate whether each material, system or method listed is relevant to your study. If you are not sure if a list item applies to your research, read the appropriate section before selecting a response.

Materials & experimental systems

n/a	Involved in the study
☐	☒ Antibodies
☐	☒ Eukaryotic cell lines
☒	☐ Palaeontology and archaeology
☐	☒ Animals and other organisms
☒	☐ Clinical data
☒	☐ Dual use research of concern

Methods

n/a	Involved in the study
☒	☐ ChIP-seq
☐	☒ Flow cytometry
☒	☐ MRI-based neuroimaging

Antibodies

Antibodies used	The following antibodies were used for western blot or immunoprecipitation: anti-SigA (BioLegend, 663205; 1:1,000 dilution in immunoblotting), anti-Rv0884c (ABclonal, 1:1,000 dilution in immunoblotting), anti-p-S6K1 (CST, 9234; 1:1,000 dilution in immunoblotting), anti-S6K1 (CST, 9202; 1:1,000 dilution in immunoblotting), anti-mTOR (CST, 2983; 1:100 dilution in immunostaining), anti-GAPDH (Abcam, 128915; 1:10,000 dilution in immunoblotting), anti-Sec13 (Santa Cruz, 514308; 1:1,000 dilution in immunoblotting and immunoprecipitation), anti-WDR24 (Proteintech, 20778-1-AP; 1:1,000 dilution in immunoblotting; 1:50 dilution in immunoprecipitation and immunoprecipitation), anti-β-Actin (Sigma, A5441; 1:5000 dilution in immunoblotting and immunoprecipitation), anti-GAPDH (Sigma, G9545; 1:5000 dilution in immunoblotting and immunoprecipitation), Goat Anti-Rabbit IgG Antibody, Peroxidase Conjugated (Sigma, AP132P, 1:5000 dilution in immunoblotting and immunoprecipitation). The following antibodies were used for immunostaining: anti-LAMP2 (Thermo Fisher, MA1-205; 1:100 dilution in immunostaining), Donkey anti-Mouse IgG (H+L) Highly Cross-Adsorbed Secondary Antibody, Alexa Fluor 488 (Thermo Fisher, A-21202, 1:100 dilution in immunostaining), Goat anti-Rabbit IgG (H+L) Cross-Adsorbed Secondary Antibody, Alexa Fluor 568 (Thermo Fisher, A-11011, 1:100 dilution in immunostaining), Goat anti-Mouse IgG (H+L) Highly Cross-Adsorbed Secondary Antibody, Alexa Fluor 568 (Thermo Fisher, A-11031, 1:100 dilution in immunostaining) and Goat anti-Rabbit IgG (H+L) Highly Cross-Adsorbed Secondary Antibody, Alexa Fluor 488 (Thermo Fisher, A-11034, 1:100 dilution in immunostaining). The following antibodies were used for flow cytometry: anti-IFNγ (Biolegend, XMG1.2, 1:200), anti-P-STAT4(Tyr693) (Invitrogen, 4LURPIE, 1:200), anti-CD3e (eBioscience, 145-2C11, 1:200), anti-CD4 (Biolegend, GK1.5, 1:200), anti-P-STAT1(Ser727) (Invitrogen, Stat1S727-C6, 1:200), anti-CD44 (Invitrogen, IM7, 1:200), anti-EOMES (Invitrogen, Dan11mag, 1:200), anti-CD69 (Invitrogen, H1.2F3, 1:20), anti-T-bet (Invitrogen, 4B10, 1:200), anti-IL-17A (Invitrogen, eBio17B7, 1:200), P-S6 Ribosomal (S235/236) (Cell Signaling, 8520S, 1:200), anti-CD25 (Invitrogen, PC61.5, 1:200), anti-Granzyme B (Invitrogen, NGZB, 1:200), anti-CD8a (Invitrogen, 53-6.7, 1:200), anti-TNF-α (BD, MP6-XT22, 1:200), TB10.44–11 H-2Kb tetramer (Proimmune, F764-2A-G, 1:20), 32a309-319/Db tetramer (MBL, TS-M549-2, 1:20)
Validation	The commercial antibodies are well used and reported in lots of previous publications. The specificity of the anti-Rv0884c antibody via western blot was analysed using purified recombinant Rv0884c107-376aa protein (10ng, 5ng, 1ng, 500pg). The anti-Rv0884c antibody can bind and detect Rv0884c107-376aa antigen at 500pg, indicating the high-affinity and specificity of this antibody, as shown in Extended Data Figure 1d. According to the Extended Data Figure 1f, signals were compared between Rv0884c_cKDTet strains treated with or without ATc samples, weak expected banding on a western blotting in the Rv0884c_cKDTet strain treated with ATc sample further indicated the specificity of the anti-Rv0884c antibody.<br>Validation statement for anti-SigA https://www.biolegend.com/en-us/products/direct-blot-hrp-anti-eme-coli-em-rna-sigma-70-antibody-13486<br>Validation statement for anti-p-S6K1 https://www.cellsignal.cn/product/productDetail.jsp?productId=9234<br>Validation statement for anti-S6K1 https://www.cellsignal.cn/product/productDetail.jsp?productId=9202<br>Validation statement for anti-mTOR https://www.cellsignal.cn/product/productDetail.jsp?productId=2983<br>Validation statement for anti-GAPDH https://www.abcam.cn/products/primary-antibodies/gapdh-antibody-epr6256-loading-control-ab128915.html<br>Validation statement for anti-Sec13 https://www.scbt.com/p/sec13-antibody-f-6?requestFrom=search<br>Validation statement for anti-WDR24 https://www.ptgcn.com/products/WDR24-Antibody-20778-1-AP.htm<br>Validation statement for anti-β-Actin https://www.sigmaaldrich.cn/CN/zh/product/sigma/a5441<br>Validation statement for anti-GAPDH https://www.sigmaaldrich.cn/CN/zh/product/sigma/g9545<br>Validation statement for anti-LAMP2 https://www.thermofisher.cn/cn/zh/antibody/product/LAMP2-Antibody-clone-H4B4-Monoclonal/MA1-205<br>Validation statement for anti-IFNγ https://www.biolegend.com/en-us/products/apc-anti-mouse-ifn-gamma-antibody-993<br>Validation statement for anti-P-STAT4(Tyr693) https://www.thermofisher.cn/cn/zh/antibody/product/Phospho-STAT4-Tyr693-Antibody-clone-4LURPIE-Monoclonal/12-9044-42<br>Validation statement for anti-CD3e https://www.thermofisher.cn/antibody/primary/query/145-2C11<br>Validation statement for anti-CD4 https://www.biolegend.com/en-us/products/apc-cyanine7-anti-mouse-cd4-antibody-1964<br>Validation statement for anti-P-STAT1(Ser727) https://www.thermofisher.cn/cn/zh/antibody/product/Phospho-Stat1-Ser727-Antibody-clone-Stat1S727-C6-Recombinant-Monoclonal/MA5-37074<br>Validation statement for anti-CD44 https://www.thermofisher.cn/cn/zh/antibody/product/CD44-Antibody-clone-IM7-Monoclonal/25-0441-82<br>Validation statement for anti-EOMES https://www.thermofisher.cn/cn/zh/antibody/product/EOMES-Antibody-clone-Dan11mag-Monoclonal/12-4875-82<br>Validation statement for anti-CD69 https://www.thermofisher.cn/cn/zh/antibody/product/CD69-Antibody-clone-H1-2F3-Monoclonal/11-0691-82<br>Validation statement for anti-T-bet https://www.thermofisher.cn/cn/zh/antibody/product/T-bet-Antibody-clone-eBio4B10-4B10-Monoclonal/50-5825-82<br>Validation statement for anti-IL-17A https://www.thermofisher.cn/cn/zh/antibody/product/IL-17A-Antibody-clone-eBio17B7-Monoclonal/17-7177-81<br>Validation statement for P-S6 Ribosomal (S235/236) https://www.cellsignal.cn/product/productDetail.jsp?productId=8520<br>Validation statement for anti-CD25 https://www.thermofisher.cn/cn/zh/antibody/product/CD25-Antibody-clone-PC61-5-

Monoclonal/25-0251-82
Validation statement for anti-Granzyme B https://www.thermofisher.cn/cn/zh/antibody/product/Granzyme-B-Antibody-clone-NGZB-Monoclonal/25-8898-82
Validation statement for anti-CD8a https://www.thermofisher.cn/cn/zh/antibody/product/CD8a-Antibody-clone-53-6-7-Monoclonal/45-0081-82
Validation statement for anti-TNF-α
https://www.bdbiosciences.com/zh-cn/products/reagents/flow-cytometry-reagents/research-reagents/single-color-antibodies-ruo/bv421-rat-anti-mouse-tnf.563387

Eukaryotic cell lines

Policy information about cell lines and Sex and Gender in Research

Cell line source(s)	HEK293T cells (ATCC CRL-3216) and jurkat cell line (ATCC TIB-152) were obtained from the American type culture collection (ATCC), and iBMDMs were provided by Prof. F. Shao (National Institute of Biological Sciences, Beijing), as described in Materials and Methods section (Cell lines part).
Authentication	Cell lines purchased from commercial vendors, ATCC have been authenticated by the commercial vendor using short tandem repeat (STR) analysis. The iBMDMs cell line used was not authenticated.
Mycoplasma contamination	All the cells were routinely tested for contamination by mycoplasma.
Commonly misidentified lines (See ICLAC register)	No commonly misidentified cell lines were used in this study.

Animals and other research organisms

Policy information about studies involving animals; ARRIVE guidelines recommended for reporting animal research, and Sex and Gender in Research

Laboratory animals	Cd4Cre, WDR24fl/fl, Rag1-/- and Ifngfl/fl, TB10Rg3 TCR specific mice on C57BL/6 genetic background were purchased from the Cyagen Biosciences. Female 6-8 weeks old SPF C57BL/6 mice were purchased from Slaccas. 6-8 weeks old female C57BL/6 mice and TB10Rg3 TCR specific mice were used for CD8+T cell separation and BMDM extraction. 6-8 weeks old C57BL/6 female mice, CD4CreIfngfl/fl female mice, CD4CreWDR24fl/fl and Rag1-/- female mice were used for infection. All the mice were bred in specific pathogen-free conditions at the Shanghai Pulmonary Hospital Laboratory Animal Center (temperature is between 20-26 ?, relative humidity is between 50% and 60%, the light intensity in the feeding room is 15-20lx with a light and dark alternation of 12h/12h) and were fed with 25kGy irradiated feed (SLAC, P1101F) in accordance with hospital guidelines.
Wild animals	The study did not involve wild animals.
Reporting on sex	All mice were age-, weight, and sex-matched in each experiment.
Field-collected samples	The study did not involve samples collected from the field.
Ethics oversight	All animal experiments were reviewed and approved by the Animal Experiment Administration Committee of Shanghai Pulmonary Hospital and were conducted in accordance with the National Institutes of Health (NIH) Guidelines for the Care and Use of Laboratory Animals.

Note that full information on the approval of the study protocol must also be provided in the manuscript.

Flow Cytometry

Plots

Confirm that:

☒ The axis labels state the marker and fluorochrome used (e.g. CD4-FITC).

☒ The axis scales are clearly visible. Include numbers along axes only for bottom left plot of group (a 'group' is an analysis of identical markers).

☒ All plots are contour plots with outliers or pseudocolor plots.

☒ A numerical value for number of cells or percentage (with statistics) is provided.

Methodology

Sample preparation	To analyze surface markers, cells were stained in PBS (Gibco) containing 2% (wt/vol) BSA (Sigma). Surface proteins were stained for 30min on ice. For phospho-flow cytometry analysis, to preserve the fluorescent proteins, cells were fixed with 2% paraformaldehyde for 30min at room temperature, followed by permeabilization with 90% ice-cold methanol for 30min and then stained in 1× permeabilization buffer (eBioscience) for 30min at room temperature. Transcription factor staining was performed with FOXP3/transcription factor staining buffers, according to the manufacturer's instructions (eBioscience).

	Intracellular staining for cytokines was performed with a fixation/ permeabilization kit (BD Biosciences). In brief, cells were activated using Cell Stimulation Cocktail (eBioscience, 00-4975-93) and then stained with surface markers for 30 min, fixed, permeabilized using fixation/ permeabilization kit (BD Biosciences), and stained with anti-IFN-γ (Biolegend, XMG1.2), anti-IL-17A (Invitrogen, eBio17B7), anti-Granzyme B (Invitrogen, NGZB) and anti-TNF-α (BD, MP6-XT22) antibodies. Cells were washed twice and the frequency of IFN-γ+ CD8+T cells, IL-17A+ CD8+T cells, Granzyme B+ CD8+T cells and TNF-α+ CD8+T cells was determined by FCM. The determination of mitochondrial mass was conducted using flow cytometry according to the instructions of the manufacturer (Thermo Fisher, M7514). FMO controls were used for accurately discriminating positive versus negative signals of CD69 and Eomes staining.
Instrument	CytoFLEX LX and BD AriaIII
Software	Analyzer:BD FACSDiva<br>Data analysis: cytoflex cytexpert
Cell population abundance	all singlets were included
Gating strategy	FSC-H vs. FSC-A density plot gating was performed to identify singlets and SSC-A vs. FSC-A used to gate on intact cells and gate boundaries were defined based on untreated cells and then target cells quantified by fluorescence.

☒ Tick this box to confirm that a figure exemplifying the gating strategy is provided in the Supplementary Information.
