# Supplementary figures and images for: Mycobacterium tuberculosis produces d-serine under hypoxia to limit CD8+ T cell-dependent immunity in mice
Source: Nat Microbiol. 2024 May 28;9(7):1856–72. doi: 10.1038/s41564-024-01701-1 (PMC11222154; doi:10.1038/s41564-024-01701-1)

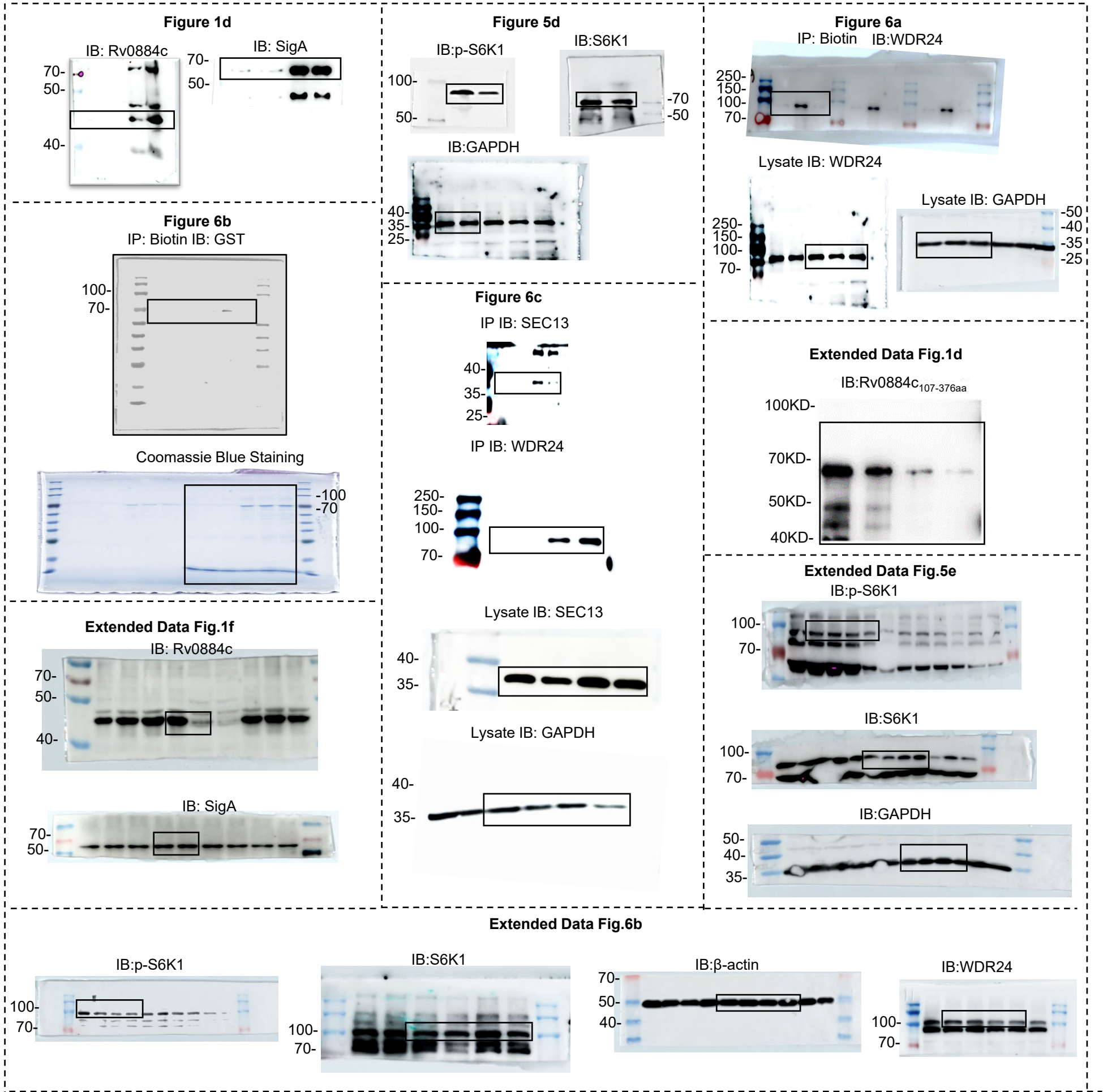

Full immunoblots shown in the manuscript

Supplement: Supplementary file 16 — Unprocessed western blots and/or gels. [file 41564_2024_1701_MOESM16_ESM.pdf]
